# Dysregulation of xenobiotic metabolism and mitochondrial dysfunction exacerbate acetaminophen-induced hepatotoxicity in human antigen R-deficient male mice

**DOI:** 10.1016/j.tox.2026.154480

**Published:** 2026-04-28

**Authors:** Natalie Eppler, Elizabeth Jones, Forkan Ahamed, Naren Raja, Jephte Y. Akakpo, Margitta Lebofsky, Lily He, Ira Vats, Priyanka Ghosh, Yifan Yu, Kaitlyn Thomas, Colin McCoin, John Thyfault, Xiaoqing Wu, Liang Xu, Wei Cui, Rong Wang, Hartmut Jaeschke, Yuxia Zhang

**Affiliations:** a Department of Pharmacology, Toxicology and Therapeutics, University of Kansas Medical Center, KS 66160, USA; b Department of Oral and Craniofacial Sciences, School of Dentistry, University of Missouri-Kansas City, Kansas City, MO 64108, USA; c Departments of Cell Biology and Physiology, KU Diabetes Institute, and Kansas Center for Metabolism and Obesity Research, University of Kansas Medical Center, KS 66160, USA; d Department of Molecular Biosciences, University of Kansas, Lawrence, KS 66045, USA; e Department of Pathology, University of Kansas Medical Center, KS 66160, USA

**Keywords:** Acetaminophen hepatotoxicity, Human antigen R, Gene knockout, Male mouse, Xenobiotic metabolism, Mitochondrial function

## Abstract

Acetaminophen (APAP) overdose is a leading cause of acute liver failure worldwide. The RNA-binding protein Human antigen R (HuR) is a multifunctional post-transcriptional regulator that plays a pivotal role in cellular stress responses, including those triggered by APAP toxicity. This study investigated the protective role and mechanisms of HuR in APAP-induced hepatotoxicity in male mice. Hepatocyte-specific *HuR*-deficient (*HuR*^Hep−/−^) mice on a C57BL/6 N background and wild-type (WT) littermates were treated with 200 mg/kg APAP, and liver tissues were collected at 2, 6, and 24 h post-treatment. APAP exposure increased hepatic *HuR* mRNA expression and induced both HuR cleavage and the formation of a higher-molecular-weight HuR species, which correlated with injury severity. Compared with WT controls, *HuR*^Hep−/−^ mice exhibited markedly increased susceptibility to hepatotoxicity at both 2 and 6 h. Metabolite profiling revealed altered APAP metabolism and reduced expression of glutathione S-transferases (Gsts) in *HuR*^Hep−/−^ livers, consistent with impaired detoxification and increased APAP-protein adduct formation. Fourier-transform infrared (FTIR) spectroscopy further identified early biochemical alterations between genotypes as early as 2 h after APAP exposure. *HuR* deficiency also resulted in pronounced mitochondrial structural abnormalities and dysfunction, accompanied by reduced expression of mitochondrial fission and fusion proteins (Drp1 and Mfn2), increased mitochondrial protein release, and enhanced hepatocyte death. Mechanistically, HuR inhibition and overexpression studies demonstrated its regulatory role in genes involved in detoxification and mitochondrial integrity. Ribonucleoprotein immunoprecipitation (RNP-IP) confirmed direct binding of HuR to mRNAs encoding mitochondrial dynamics proteins (Drp1, Mfn2), detoxification enzymes (Gsta4, Gstm6), and antioxidant regulators (Nrf2, Gclc, Gclm). Collectively, these findings identify hepatocyte HuR as a critical regulator of xenobiotic metabolism and mitochondrial function and establish the essential role of HuR in early protection against APAP-induced hepatotoxicity.

## 1. Introduction

Globally, acetaminophen (APAP), also known as paracetamol, is among the most widely used analgesic and antipyretic medications (Chidiac et al. 2023). Due to its narrow therapeutic index and widespread presence in over-the-counter medications, APAP is the leading cause of acute liver failure in the United Kingdom and the United States (Bernal and Wendon, 2013). Early administration of the sulfhydryl donor, N-acetylcysteine (NAC), is key to detoxification and patient survival following an APAP overdose (Green et al. 2013). However, APAP overdose patients often present late to the clinic, limiting the efficacy of current intervention strategies (Chidiac et al. 2023). To improve current intervention strategies, a greater mechanistic understanding of APAP-induced hepatotoxicity is warranted.

The liver plays a central role in the metabolism and elimination of APAP. At therapeutic doses, APAP is primarily detoxified through glucuronidation and sulfation to form non-reactive metabolites (McGill and Jaeschke, 2013). However, at supratherapeutic doses, the sulfation pathway becomes saturated, leading to increased formation of the hepatotoxic metabolite N-acetyl-p-benzoquinone imine (NAPQI) (Ramachandran and Jaeschke 2019a). Rapid detoxification of NAPQI through conjugation with glutathione (GSH) is essential to prevent hepatic injury (Mitchell et al. 1973). The resulting APAP conjugates are subsequently eliminated from hepatocytes via apical and basolateral membrane transport proteins (McGill and Jaeschke, 2013). Experimental studies targeting various drug-metabolizing enzymes (DMEs) and membrane transporters have revealed complex and overlapping mechanisms governing APAP detoxification and disposition (Henderson et al. 2000; Zamek-Gliszczynski et al. 2006). Although the number of DMEs is relatively limited compared with the vast array of xenobiotics they process, modulation of their expression through induction or repression has profound effects on xenobiotic half-life, activation, and tissue distribution. Nuclear receptors such as pregnane X receptor (PXR) and constitutive androstane receptor (CAR) are well established transcriptional regulators of DMEs in this context (Willson and Kliewer, 2002). In contrast, the post-transcriptional regulation of DME expression remains poorly understood. Tight control of mRNA stability by RNA-binding proteins is a key mechanism that enables cells to rapidly adapt to environmental changes. Human antigen R (HuR; ELAVL1) is a ubiquitously expressed post-transcriptional regulator that binds adenine- and uridine-rich elements (AREs) and U-rich sequences within the 3′ untranslated regions and intronic regions of target mRNAs (Lebedeva et al. 2011; Lopez de Silanes et al., 2004). While HuR binding primarily stabilizes mRNAs, it also regulates mRNA export, translation, and, in some cases, mRNA decay (Eppler et al. 2025; Galban et al. 2008). In addition, HuR has nuclear functions, including the regulation of alternative splicing and polyadenylation (Akaike et al. 2014). Accordingly, a major objective of this study was to elucidate the role of HuR in the post-transcriptional regulation of hepatic APAP metabolism.

Mitochondria are particularly susceptible to NAPQI-mediated toxicity, and interventions that mitigate mitochondrial injury provide significant protection against APAP-induced hepatotoxicity (Ramachandran et al. 2018). Within mitochondria, covalent binding of NAPQI to nucleophilic proteins disrupts the respiratory chain, enhances reactive oxygen species generation, dissipates mitochondrial membrane potential, and ultimately triggers opening of the mitochondrial permeability transition (MPT) pore, leading to necrotic cell death (Ramachandran and Jaeschke 2019b). Moreover, disturbances in mitochondrial morphology and dynamics impair respiratory efficiency (Donnelly et al. 1994) and increase mitochondrial susceptibility to MPT pore opening (Picard et al. 2013). In the liver, HuR is activated in response to oxidative stress and confers cyto-protection in murine hepatocytes during ischemia reperfusion injury, in part through induction of the antioxidant enzyme heme oxygenase-1 (HO-1) (Dery et al. 2020). Similarly, HuR protects against diet-induced mitochondrial dysfunction by regulating expression of key electron transport chain components, including cytochrome c (Cytc), NADH:ubiquinone oxidoreductase subunit B6 (Ndufb6), and ubiquinol–cytochrome c reductase binding protein (Uqcrb) (Zhang et al. 2020). Recent evidence also indicates that hepatocyte HuR protects against APAP-induced liver injury by promoting hepatocyte proliferation, autophagy, and antioxidant defense following overdose (Lu et al. 2024). However, whether the exacerbated liver injury observed in hepatocyte-specific *HuR*-deficient (*HuR*^Hep−/−^) mice results, at least in part, from mitochondrial dysfunction associated oxidative stress remains unclear. Therefore, the second aim of this study was to evaluate mitochondrial structural and functional integrity in *HuR*^Hep−/−^ livers under normal condition and over the time course following APAP overdose.

Fourier transform infrared (FTIR) spectroscopy is a label-free and highly sensitive technique for detecting subtle biomolecular alterations in biological samples (Baker et al. 2014). By measuring infrared light absorption, FTIR generates unique molecular fingerprints, particularly within the fingerprint region (1800–900 cm^−1^), which corresponds to vibrational transitions of proteins, lipids, carbohydrates, and nucleic acids (Wang and Wang, 2021). Our previous studies demonstrated that FTIR-based biomolecular profiling can effectively differentiate oral squamous cell carcinoma from benign tissues (Wang et al. 2021), predict malignant transformation risk in precancerous oral lesions (Wang et al. 2025), and identify biochemical signatures associated with tumor treatment responses (Ly et al. 2025). Despite its proven utility in oncology, the application of FTIR spectroscopy in detecting early biochemical alterations in liver tissues following drug-induced injury remains unexplored. Therefore, the third aim of this study was to employ FTIR spectroscopy to characterize subtle molecular differences between wild-type (WT) and *HuR*^Hep−/−^ livers following APAP overdose, thereby assessing its potential as a sensitive, non-destructive analytical tool for early detection of molecular perturbations in liver toxicology research.

In the present study, we demonstrate that the disposition of GSH-conjugates of APAP metabolites is markedly altered in *HuR*^Hep−/−^ mice, accompanied by dysregulation of glutathione S-transferases (Gsts), key phase II DMEs. Consistent with these findings, FTIR analysis revealed early alterations in hepatic biochemical composition in *HuR*-deficient livers after APAP exposure. In addition, *HuR*^Hep−/−^ livers exhibited reduced mitochondrial abundance and disrupted mitochondrial morphology, along with reduced expression of mitochondrial fusion and fission proteins (Mfn2 and Drp1), impaired respiratory capacity, and increased ROS production. Mechanistically, HuR inhibition and overexpression indicate that HuR regulates genes governing detoxification and mitochondrial integrity. Ribonucleoprotein immunoprecipitation (RNP-IP) further confirmed direct binding of HuR to mRNAs encoding mitochondrial dynamics proteins (Drp1, Mfn2), detoxification enzymes (Gsta4, Gstm6), and antioxidant regulators (Nrf2, Gclc, Gclm). Together, these findings support a protective role for HuR in maintaining hepatic function during acute APAP-induced injury and reveal multiple regulatory mechanisms underlying this effect. Collectively, our study provides new insight into the molecular networks governed by hepatocyte HuR, highlighting its essential role in xenobiotic metabolism, mitochondrial integrity, and early protection against APAP-induced hepatotoxicity.

## 2. Materials and methods

### 2.1. Animal studies

*HuR*^flox/flox^ mice (JAX stock #: 021431) and Alb-Cre mice (JAX stock #: 003574) were obtained from Jackson Laboratory (Bar Harbor, ME, USA). Hepatocyte *HuR* knockout (*HuR*^Hep−/−^ represents HuR^flox/flox^; Alb-Cre positive) and their littermate wild-type controls (WT represents HuR^flox/flox^; Alb-Cre negative) were generated by crossbreeding *HuR*^flox/flox^ mice with Alb-Cre mice and subsequently backcrossing into the C57BL/6 N genetic background for 10 generations. Mice were housed in a virus-free facility with a 12 h light/dark cycle (lights on from 6 a.m.–6 p.m.) and maintained at a temperature of 25°C, with ad libitum access to food and water. Male mice aged 8–10 weeks were used for the experiments (n = 5–6/group) and randomly assigned to experimental groups. Prior to APAP administration, mice were fasted overnight. APAP from Sigma-Aldrich (St. Louis, MO, USA) was dissolved in warm saline and administered intraperitoneally at a dose of 200 mg/kg. All animal experiments were performed in accordance with the NIH Guide for the Care and Use of Laboratory Animals and reported following the ARRIVE guidelines. Animal protocol was approved by the Institutional Animal Care and Use Committee (ICAUC) at the University of Kansas Medical Center.

### 2.2. Histopathology and immunohistochemistry

Liver tissues were fixed in 10% formalin in PBS (pH 7.4) for 24 h, processed, paraffin-embedded, sectioned into 5 μm slices, and stained with hematoxylin and eosin (H&E) for histopathological evaluation. The extent of liver injury was assessed by a pathologist Dr. Wei Cui who was blinded to genotype and treatment group information. Terminal deoxynucleotidyl transferase dUTP nick end labeling (TUNEL) staining was performed on rehydrated liver sections using the In Situ Cell Death Detection Kit (Roche, 11684809910) to assess cell death. For immunohistochemistry (IHC), antigen retrieval was performed on rehydrated sections and sodium citrate buffer (10 mM, pH 6.0) in a pressure cooker at high pressure for 5 min was used for F4/80, while Tris-EDTA buffer (10 mM, pH 9.0) at high pressure for 10 min was used for PCNA and Ly6g. Endogenous peroxidase activity was blocked with 0.3% H_2_O_2_, and non-specific binding was prevented with 10% normal horse serum. Sections were incubated overnight at 4°C with primary antibodies: F4/80 (Cell Signaling Technology, 70076), PCNA (Cell Signaling Technology, 13110S), and Ly6g (Cell Signaling Technology, 87048). Antibody binding was detected using ImmPRESS peroxidase polymer detection kits (Vector Laboratories, MP-7402) and ImmPACT DAB substrate (Vector Laboratories, SK-4105). Sections were counterstained with hematoxylin. An anti-3-Nitrotyrosine antibody (Abcam, ab61392) was used for immunofluorescence staining. For image acquisition, five fields per slide were randomly selected and imaged using an Olympus IX73 microscope. Quantification was performed using Fiji (ImageJ), and measurements from each animal were averaged to generate a single value representing one biological replicate for statistical analysis. No samples were excluded after data acquisition unless technical artifacts (e.g., tissue processing defects) were present.

### 2.3. FTIR imaging and data analysis

Paraffin-embedded liver tissues sectioned at 5 μm thickness were mounted on barium fluoride (BaF_2_) discs (25 × 2 mm; REFLEX Analytical Corp., Ridgewood, NJ, USA) for FTIR imaging. Sections were deparaffinized in histological-grade xylene (CAS 1330–20–7; Sigma-Aldrich, St. Louis, MO, USA) for 5 min × 3 at room temperature, air-dried, and stored overnight in a vacuum desiccator. FTIR images were acquired in transmission mode using a PerkinElmer Spectrum Spotlight imaging system (Spectrum One, Spotlight 300; PerkinElmer, Waltham, MA, USA) with Spectrum IMAGE software. Imaging parameters were as follows: spectral resolution, 4 cm^−1^ ; spectral range, 4000–950 cm^−1^ ; pixel size, 6.25 μm; and 16 co-added scans per pixel. Three regions of interest (ROIs), each measuring 400 × 400 μm and located near central vein regions, were selected per tissue section. Spectral preprocessing and analysis were conducted using PLS_Toolbox (Eigenvector Research Inc., Manson, WA, USA) in MATLAB R2020b (MathWorks, Natick, MA, USA). The workflow included: (1) transmission-to-absorbance conversion (A = log(1/T)); (2) selection of the fingerprint region (1800–950 cm^−1^); (3) Savitzky–Golay smoothing; (4) extended multiplicative signal correction; (5) automated weighted least-squares baseline correction; and (6) vector normalization. Principal component analysis (PCA) and Hotelling’s T^2^ versus Q-residuals plots were then applied for exploratory analysis and outlier removal (Wang et al. 2021). A representative FTIR spectrum for each ROI was obtained by averaging pixel-level spectra and used for subsequent analysis. Three animals per group was used for FTIR imaging and the animal slection was based on H&E staining and serum ALT levels that most closely reflected the average injury severity of the respective group.

### 2.4. Determination of APAP metabolites in mouse plasma by LC-MS/MS

APAP metabolites in mouse plasma were detected as described previously (Akakpo et al. 2018; Ghosh et al. 2023). Standards, including 4-acetamidophenyl β-D-glucuronide (APAP-Gluc), 4-acetaminophen sulfate (APAP-Sulf), 3-(N-acetylcysteine) acetaminophen (APAP-NAC), 3-cysteinylacetaminophen trifluoroacetic acid (APAP-Cys), acetaminophen glutathione (APAP-GSH), along with internal standards acetaminophen-d4 and acetaminophen-sulfate-d3, were sourced from Toronto Research Chemicals (Toronto, Canada). Stock solutions (1 mM) and a mixed standard solution (75 μM) were prepared in 50:50 methanol: water, with working standards (0.25–25 μM) diluted from the stock. Samples and standards were prepared on ice. Plasma from untreated mice was pooled as the blank matrix. To precipitate proteins, 20 μl of plasma or 50 μl of working standard was mixed with 90 μl of methanol containing internal standards (200 ng/ml acetaminophen-d4 and 1000 ng/ml acetaminophen-sulfate-d3). Additional water and methanol were added, followed by vortexing for 10 s and centrifugation at 13,400 × g for 10 min at 4°C. Analysis was performed using LC-MS/MS with a Waters Acquity UPLC^®^ system and Quattro Premier XE mass spectrometer in positive mode with multiple reaction monitoring (MRM). Separation was achieved on a Waters UPLC^®^ HSS T3 column (1.8 μm, 2.1 × 150 mm) at 50°C using a gradient flow of mobile phase A (6 mM ammonium acetate in water with 0.01% formic acid) and mobile phase B (methanol). The gradient transitioned from 2% to 75% B over 3.5 min, then to 98% B over 0.5 min, and held for 2 min at 0.4 ml/min. Quantification used a weighted (1/x) linear regression of analyte/internal standard peak area ratios. Internal standard acetaminophen sulfate-d3 and QuanLynx software (version 4.1) were used for MS peak integration. Detection limits were 0.250 μM for APAP-GSH, 0.125 μM for APAP-Sulf and APAP-Gluc, 0.063 μM for APAP-Cys, and 0.025 μM for APAP-NAC. Quantification was performed using internal standards to correct for extraction efficiency and instrument variability. Metabolite levels were normalized to sample volume to ensure comparability across samples. Each sample was measured in triplicate, and the mean of the three measurements was used to represent a single biological replicate (individual mouse) for statistical comparison.

### 2.5. Analysis of APAP protein adducts by high-pressure liquid chromatography (HPLC)

Liver APAP protein adducts were quantified as described previously (Akakpo et al. 2018; Ghosh et al. 2023). Briefly, Liver homogenates were filtered through a Bio-Spin 6 column to remove lower molecular weight metabolites. To release APAP-Cys, filtrates were then digested overnight with 8 U/ml Streptomyces griseus protease. Following digestion, lysates were precipitated using 40% trichloroacetic acid for 10 min on ice. The residual APAP-Cys adducts were quantified using high-performance liquid chromatography with electrochemical detection (HPLC-ECD) and were further normalized to tissue weight to account for differences in sample size. Each sample was measured in triplicate, and the mean of the three measurements was used to represent a single biological replicate (individual mouse) in the figure.

### 2.6. Hepatic mitochondrial isolation and measurement of respiration and H_2_O_2_ emission

Hepatic mitochondria were isolated as described previously (Kumari et al. 2024; McCoin et al. 2019). Briefly, liver tissue was excised, submerged in 8 ml cold isolation buffer (220 mM mannitol, 70 mM sucrose, 10 mM Tris, 1 mM EDTA, pH 7.4), and homogenized on ice. Homogenates were centrifuged at 4°C for 10 min at 1500 × g. The supernatant was strained and centrifuged again at 4°C for 10 min at 8000 × g. The mitochondrial pellet was resuspended in 6 ml isolation buffer and centrifuged at 4°C for 10 min at 6000 × g. This step was repeated using 4 ml isolation buffer containing 0.1% fatty acid-free BSA. The final pellet was resuspended in MiR05 mitochondrial respiration buffer (pH 7.1) and used for respiration studies. Mitochondrial respiration and H_2_O_2_ emission were measured simultaneously using an Oroboros O2K fluorometer (Oroboros Instruments, Austria) following established protocols (Kumari et al. 2024; McCoin et al. 2019). Experiments started with 2 mM malate, 10 mM coenzyme A, and 2.5 mM L-carnitine. Potassium pyruvate (5 mM) was added to measure basal respiration (Leak). Maximal coupled respiration (State 3) was determined with 2.5 mM adenosine diphosphate (ADP) and further complex I-driven rates were measured with 2 mM glutamate. Maximal respiratory rates (State 3S) were assessed by adding 10 mM succinate, and uncoupled respiration was measured with 0.1 mM carbonyl cyanide-p-(trifluoromethoxy) phenylhydrazone (FCCP) titrations. All data were normalized to mitochondrial protein content determined by a DC protein assay (BIO-RAD, Hercules, CA). OXPHOS capacity was calculated as State 3 respiration minus Leak, and mitochondrial redox was determined as basal H_2_O_2_ production divided by basal respiration. Data were analyzed using DatLab 7 Software. Each sample was measured in triplicate, and the mean of the three measurements was used to represent a single biological replicate (individual mouse) in the figure.

### 2.7. Electron microscopy

To assess mitochondrial morphology, transmission electron microscopy (TEM) was performed on hepatocyte mitochondria from untreated livers and from livers collected 2, 6, and 24 h after APAP overdose in WT and *HuR*^Hep−/−^ mice. For each genotype and time point, one mouse was examined, and multiple hepatocytes and mitochondria were quantified per animal. Representative animals were selected based on H&E staining and serum ALT levels that most closely reflected the average injury severity of the respective group. Liver tissue pieces (approximately 1 ×5 mm) were fixed with 2% glutaraldehyde in 0.1 M cacodylate buffer for 2 h at room temperature with gentle shaking, followed by overnight fixation at 4 °C. Tissues were then post-fixed in 1% osmium tetroxide (OsO_4_) and embedded in Embed-812 resin. Ultrathin sections were mounted on grids and stained with uranyl acetate and lead citrate. Sections were examined using a JEOL JEM-1400 transmission electron microscope equipped with a LaB_6_ gun (JEOL, Tokyo, Japan). For image acquisition, ten fields per section were randomly selected and captured digitally. Mitochondrial features were quantified using Fiji (ImageJ) software. Measurements were performed at the organelle level and are presented as within-animal distributions.

### 2.8. Biochemical measurements of GSH/GSSG and alanine aminotransferase (ALT)

Total liver GSH levels were determined using a modified Tietze assay (Jaeschke and Mitchell, 1990). In brief, snap-frozen livers were homogenized on ice in 3% sulfosalicylic acid containing 0.1 mM EDTA. To measure liver GSH, one aliquot of liver homogenate was added to 0.01 N HCl, centrifuged and the supernatant was further diluted with 100 mM potassium phosphate buffer (KPP). To measure GSSG, another aliquot was added to 10 mM N-ethylmaleimide (NEM) in KPP to trap GSH. The residual NEM was removed with a C18 SepPack column and GSSG was determined by the Tietze assay using dithionitrobenzoic acid. Plasma ALT was measured spectrophotometrically with an ALT reagent kit according to the manufacturer’s instructions (Pointe Scientific, Canton, MI).

### 2.9. RNA isolation and real-time PCR

Total RNA was isolated using TRIzol reagent (Invitrogen), as described previously (Magee et al. 2020; Zou et al. 2018). cDNA was synthesized from Isolated RNA using the Applied Biosystems High-Capacity cDNA Reverse Transcription Kit (Fisher Scientific, Hampton, NH, USA). Real-time PCR was performed using Applied Biosystems SYBR Green PCR Master Mix (Fisher Scientific, Hampton, NH, USA). The primer sequences used for PCR amplification can be found in Supplementary Table 1. The cDNA from each individual animal was analyzed by qPCR. The amplification results were quantified by measuring the threshold cycle (Ct) value and normalized to the expression of the housekeeping gene hypoxanthine guanine phosphoribosyl transferase 1 (*Hprt1*), which served as an internal reference to control for sample-to-sample variation in RNA input and reverse transcription efficiency. Relative gene expression was calculated using the 2−ΔΔCt method, and results are presented as fold change relative to the control group.

### 2.10. RNA sequencing data analysis

Liver transcriptome profiles from *HuR*^Hep−/−^ and WT fed a normal chow diet were analyzed using the RNA sequencing (RNA-seq) data set GSE287727, recently deposited to GEO by our group. Analysis was conducted with CLC Genomics Workbench software (Qiagen, Redwood City, CA, USA). Illumina reads were aligned to the *Mus musculus* C57BL/6 genome refseq_GRCm38.p6 using local read and reverse strand alignment. Reads were visualized, and differentially expressed genes (DEGs) were identified based on an adjusted p-value of ≤ 0.05 and a fold change ≥ 1.5 relative to controls. DEGs with an RPKM value < 0.01 (~10 total counts) in either of the *HuR*^Hep−/−^ and WT groups were excluded from further analysis. Canonical pathway enrichment was determined using Ingenuity Pathway Analysis (IPA) software (Qiagen, Redwood City, CA, USA), with pathways considered significant if they had a z-score ≥ 2.

### 2.11. Protein isolation and immunoblotting

Mouse liver protein lysates were prepared by homogenizing tissues in RIPA buffer (20 mM Tris-HCl (pH 7.5), 150 mM NaCl, 1 mM Na2EDTA, 1 mM EGTA, 1% NP-40, 1% sodium deoxycholate, 2.5 mM sodium pyrophosphate, 1 mM beta-glycerophosphate, 1 mM Na_3_VO_4_, 1 μg/ml leupeptin) with protease inhibitors (Fisher Scientific PI78410, Hampton, NH, USA). After centrifugation the protein-containing supernatant was collected for whole protein analysis. Cytoplasmic and mitochondrial protein fractions were isolated as described (Adelusi et al. 2022). Liver tissue was homogenized in ice-cold mitochondrial isolation buffer (220 mM mannitol, 70 mM sucrose, 2.5 mM HEPES, 10 mM EDTA, 1 mM EGTA, 0.1% BSA, pH 7.4) using a Teflon pestle. Homogenates were centrifuged at 2500 g for 10 min at 4° C to remove nuclei and debris. The supernatant was centrifuged again at 20,000 g for 10 min at 4°C. The cytoplasmic protein was collected from the supernatant, and the mitochondrial pellet was washed and centrifuged again. Protein concentrations were measured using the DC protein assay (BIO-RAD, Hercules, CA, USA). Protein samples were mixed with loading buffer and resolved on polyacrylamide gels by electrophoresis and transferred to nitrocellulose membranes. Membranes were blocked with 5% skim milk for 1 h at room temperature, then incubated with primary antibodies (1:1000 dilution) either overnight at 4°C or for 2 h at room temperature. Primary antibodies included rabbit anti-AIF (CST, 4642S), rabbit anti-Bax (CST, 2772S), rabbit anti-Smac (CST, D5S3R), mouse anti-HuR (Santa Cruz, sc-5261), rabbit anti-Cyp2e1 (Abcam, Ab28146), rabbit anti-pJNK (CST, 4668 P), rabbit anti-JNK (CST, 9252 T), rabbit anti-Caspase-3 (CST, 9665), rabbit anti-cyclin D1 (CST, 2978S), mouse anti-GAPDH (Sigma, G8795), mouse anti-α-tubulin (Sigma, T6199), mouse anti-β-actin (Sigma, A2228), and rabbit anti-VDAC (CST, D73D12). Following primary antibody incubation, membranes were washed and incubated with the corresponding horseradish peroxidase-conjugated secondary antibody at room temperature for 1 h. Antibody binding was visualized using either Super Signal West Pico Plus Chemiluminescent Substrate (Fisher, PI34580) or Super Signal West Femto Chemiluminescent Substrate (Fisher, PI34094). Images were captured using an Odyssey XF imaging system (LI-COR, Lincoln, NE). β-Actin, GAPDH, and VDAC were used as loading controls, and protein band densitometry was performed using Image Studio Lite software. Protein expression levels were normalized to their respective loading controls and presented as fold change relative to the control groups.

### 2.12. HuR modulation in Hepa 1–6 cells

Mouse hepatoma cell line Hepa 1–6 cells obtained from the American Type Culture Collection (ATCC, CRL-1830) were maintained in Dulbecco’s Modified Eagle Medium (DMEM) supplemented with 10% fetal bovine serum (FBS) under standard culture conditions (37 °C, 5% CO_2_). To inhibit HuR activity, cells were treated with a specific HuR inhibitor KH-39 at a final concentration of 10 μM for 48 h, with DMSO used as the vehicle control. For HuR overexpression, cells were transfected with pcDNA RFP-HuR expression plasmid for 24 h, with pcDNA RFP used as the corresponding transfection control. For each condition, cells were plated and treated in triplicate. At the end of the treatment period, cells were harvested for both RNA and protein isolation. Total RNA was extracted for subsequent qPCR analysis, and whole-cell protein lysates were prepared for Western blot analysis.

### 2.13. Validation of HuR targets by ribonucleoprotein immunoprecipitation (RNP-IP)

RNP-IP was performed in Hepa 1–6 cells to validate HuR target transcripts. Cells cultured in 100-mm dishes to approximately 6 × 10^6^ cells per dish were washed twice with ice-cold PBS and lysed in 1 ml of ice-cold lysis buffer containing 50 mM Tris-HCl (pH 8.0), 150 mM NaCl, and 1% NP-40 supplemented with protease inhibitors and RNase inhibitor on ice for at least 30 min with intermittent vortexing or gentle homogenization. After centrifugation at 12,000 × g for 20 min at 4 °C, supernatants were collected and precleared with Dynabeads Protein G (Thermo Fisher Scientific, #10003D) overnight at 4 °C with gentle rotation. The following day, supernatants were collected, and protein concentration was determined. For each immunoprecipitation, 1 mg of total protein was incubated with 1 μg of anti-HuR antibody (Santa Cruz, sc-5261) or IgG control (CST, 2729) for 3 h at 4 °C, followed by overnight incubation with 50 μL of Dynabeads Protein G at 4 °C with gentle rotation. Beads were washed three times with lysis buffer containing protease inhibitors and RNase inhibitor. RNA was then extracted from the bead-bound complexes using TRIzol reagent. In parallel, 100 μg of precleared lysate was processed for RNA isolation as the input control. Because RNA yield from immunoprecipitated samples was low, all recovered RNA was used for reverse transcription and cDNA was analyzed by qPCR. Enrichment of target transcripts in HuR immunoprecipitates relative to IgG controls was calculated after normalization to the corresponding input sample using the ΔΔCt method. The RNA-IP experiments were independently repeated twice using cells from separate culture dishes to confirm reproducible HuR binding to target mRNAs.

### 2.14. Statistical analysis

Statistical analyses were performed using GraphPad Prism 9.0 (GraphPad Software, La Jolla, CA, USA). Data are presented as mean ± SEM (standard error of the mean). Individual mice were considered biological replicates, whereas measurements obtained from multiple mitochondria or regions of interest (ROIs) within the same animal were treated as technical replicates. Statistical differences between 2 groups were analyzed using an unpaired 2-tailed Student’s t test. Comparisons involving multiple genotypes and time points were analyzed using twoway ANOVA followed by Sidak’s multiple comparisons test. For FTIR datasets with hierarchical structure (multiple ROIs per animal), statistical analyses were performed using a nested one-way ANOVA with a mixed-effects model (REML), treating mouse as a random effect to account for clustering of measurements within animals and to avoid pseudoreplication. Exact n values are provided in the figure legends. A p value *<* 0.05 was considered statistically significant.

## 3. Results

### 3.1. APAP overdose increases hepatic HuR mRNA expression and induces HuR cleavage and formation of a higher-molecular weight HuR-immunoreactive product

**Fig. 1.**
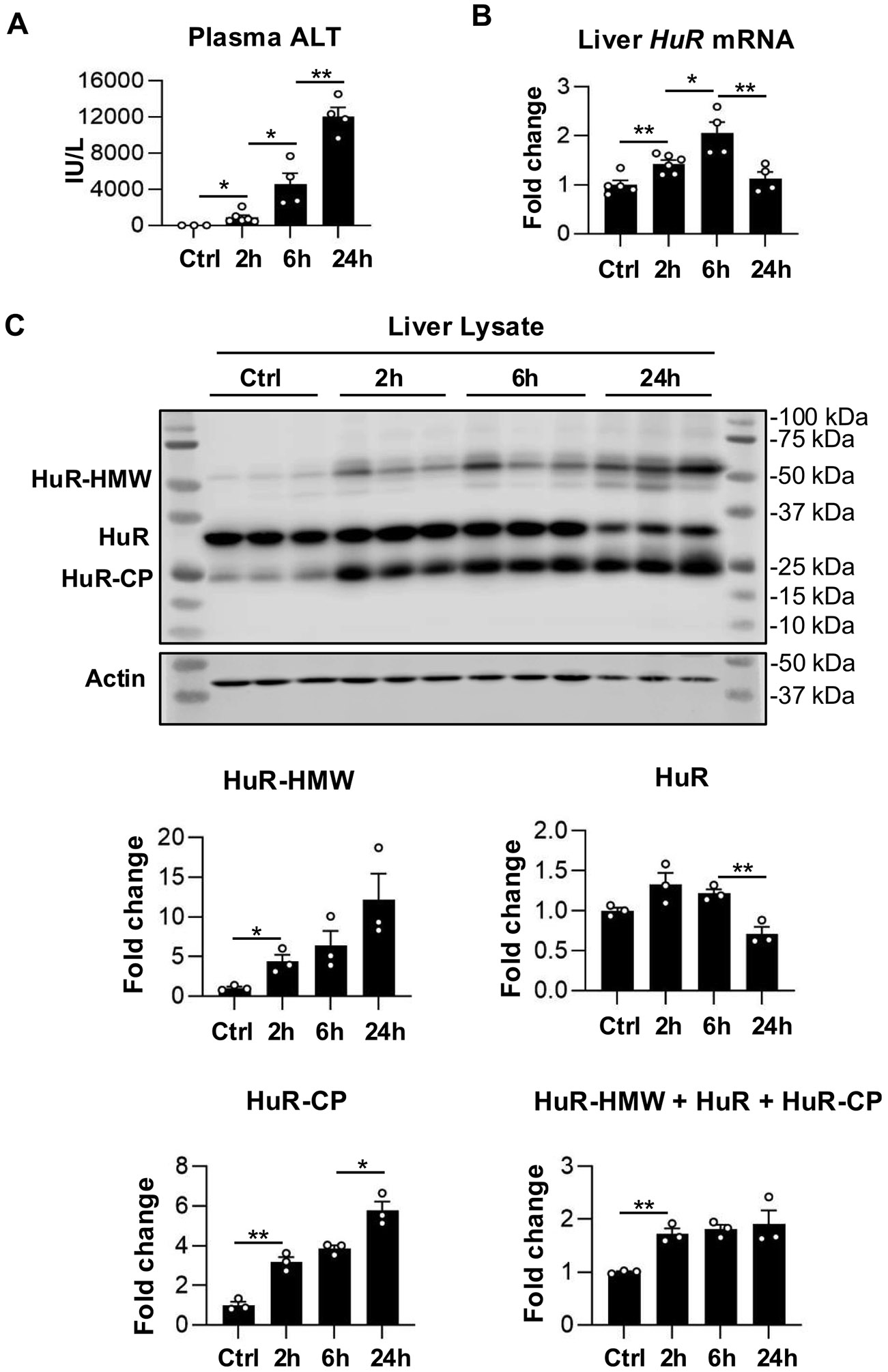
Acetaminophen overdose increases hepatic HuR mRNA expression and induces HuR cleavage and formation of a higher-molecular weight HuR-immunoreactive band in male mice. Measurements were obtained from liver and plasma samples collected at 2, 6, and 24 h following a 200 mg/kg APAP overdose. (A) Plasma alanine aminotransferase (ALT) levels following APAP overdose. (B) qPCR analysis of hepatic HuR mRNA expression. (C) Western blot analysis of HuR in whole liver lysates revealed three HuR-immunoreactive bands: a higher–molecular weight species (HuR-HMW), full-length HuR (32 kDa), and a HuR cleavage product (HuR-CP, 24 kDa). Band intensities were quantified and normalized to the loading control β-actin. Data are presented as mean ± SEM. In A-C, each data point represents an individual mouse and n = 3–6 mice per group. Comparisons involving multiple time points were analyzed using two-way ANOVA followed by Sidak’s multiple comparisons test. *p < 0.05, **p < 0.01. Abbreviations: APAP, acetaminophen; WT, wild type; ALT, aminotransferase.

Cellular stress is known to alter HuR expression, subcellular localization, post-translational protein modification, and protein proteolytic cleavage (Mazroui et al. 2008; Talwar et al. 2011). In this study, hepatic HuR expression was examined over the time course of APAP-induced hepatotoxicity in male *HuR*^flox/flox^ mice on a C57BL/6 N background following treatment with 200 mg/kg APAP. Plasma ALT levels measured at 2, 6, and 24 h after APAP overdose increased progressively (2 h vs Control, p = 0.05; 6 h vs 2 h, p = 0.04; 24 h vs 6 h, p = 0.01) (Fig. 1A) and were consistent with previous reports in C57BL/6 J mice (Bhushan et al. 2014; Duan et al. 2016). The increase in plasma ALT was accompanied by elevated hepatic *HuR* mRNA expression at 2 and 6 h following APAP treatment (2 h vs control, p = 0.005; 6 h vs 2 h, p = 0.02), whereas *HuR* mRNA levels returned to baseline by 24 h (24 h vs 6 h, p = 0.01) (Fig. 1B).

Western blot analysis of liver samples following APAP exposure revealed two additional HuR-immunoreactive bands in addition to the full-length 32 kDa HuR protein: a higher-molecular weight band (HuR-HMW) between 50 and 75 kDa and a lower-molecular weight cleavage product (HuR-CP) at approximately 25 kDa (Fig. 1C). Both HuR-HMW and HuR-CP were minimally detectable in untreated control livers but increased markedly after APAP exposure (HuR-HMW: 2 h vs control, p = 0.02; HuR-CP: 2h vs control, p = 0.002; 24h vs 6h, p = 0.02), with their abundance closely paralleling serum ALT levels. In contrast, the level of full-length 32 kDa HuR significantly decreased at 24 h (24 h vs 6 h, p = 0.007), corresponding to the time point of maximal ALT elevation. Overall, the total levels of HuR-immunoreactive bands (HuR- HMW, HuR, and HuR-CP) increased following APAP exposure (2 h vs Control, p = 0.002) (Fig. 1C). Based on its apparent molecular weight, the HuR-HMW band may represent HuR dimers or higher-order HuR-containing complexes formed under oxidative stress conditions associated with APAP-induced hepatotoxicity. Previous studies have shown that the RNA recognition motif (RRM1) and RRM1/2 domains of HuR can undergo homodimerization in vitro through a disulfide bond at cysteine 13, a mechanism that may contribute to redox-dependent regulation of HuR activity under oxidative stress conditions (Benoit et al. 2010). This is particularly relevant in APAP-induced hepatotoxicity, which is characterized by severe mitochondrial oxidative stress, reactive nitrogen species formation, and covalent protein modification by the reactive metabolite NAPQI. However, the precise identity of the HuR-HMW species observed in this study remains unclear and warrants further investigation.

Lethal cellular stress has been known to trigger caspase-mediated cleavage of HuR at aspartate 226 within its nucleocytoplasmic shuttling sequence, generating the 24-kDa HuR cleavage product that contributes to pp32/PHAP-I-mediated regulation of apoptosis (Mazroui et al. 2008). Additionally, HuR cleavage has been reported to function as a molecular switch that alters HuR affinity for target transcripts and promotes apoptosis progression (von Roretz et al. 2013). In the present study, cleaved caspase-3 was not detected at any time point following APAP exposure (Supplementary Figure 1). Because hepatocyte death after APAP overdose is well established to occur predominantly through mitochondrial dysfunction and necrotic cell death, rather than caspase-3-dependent apoptosis (Jaeschke et al. 2018; Lambrecht et al. 2024), these findings indicate that APAP-induced liver injury is associated with HuR protein modification and cleavage through caspase-3 independent mechanisms. Accordingly, further studies are warranted to define the molecular pathways responsible for HuR cleavage and modification during APAP-induced hepatotoxicity.

### 3.2. Heightened liver injury severity in HuR^Hep−/−^ mice at early time points following APAP overdose

**Fig. 2.**
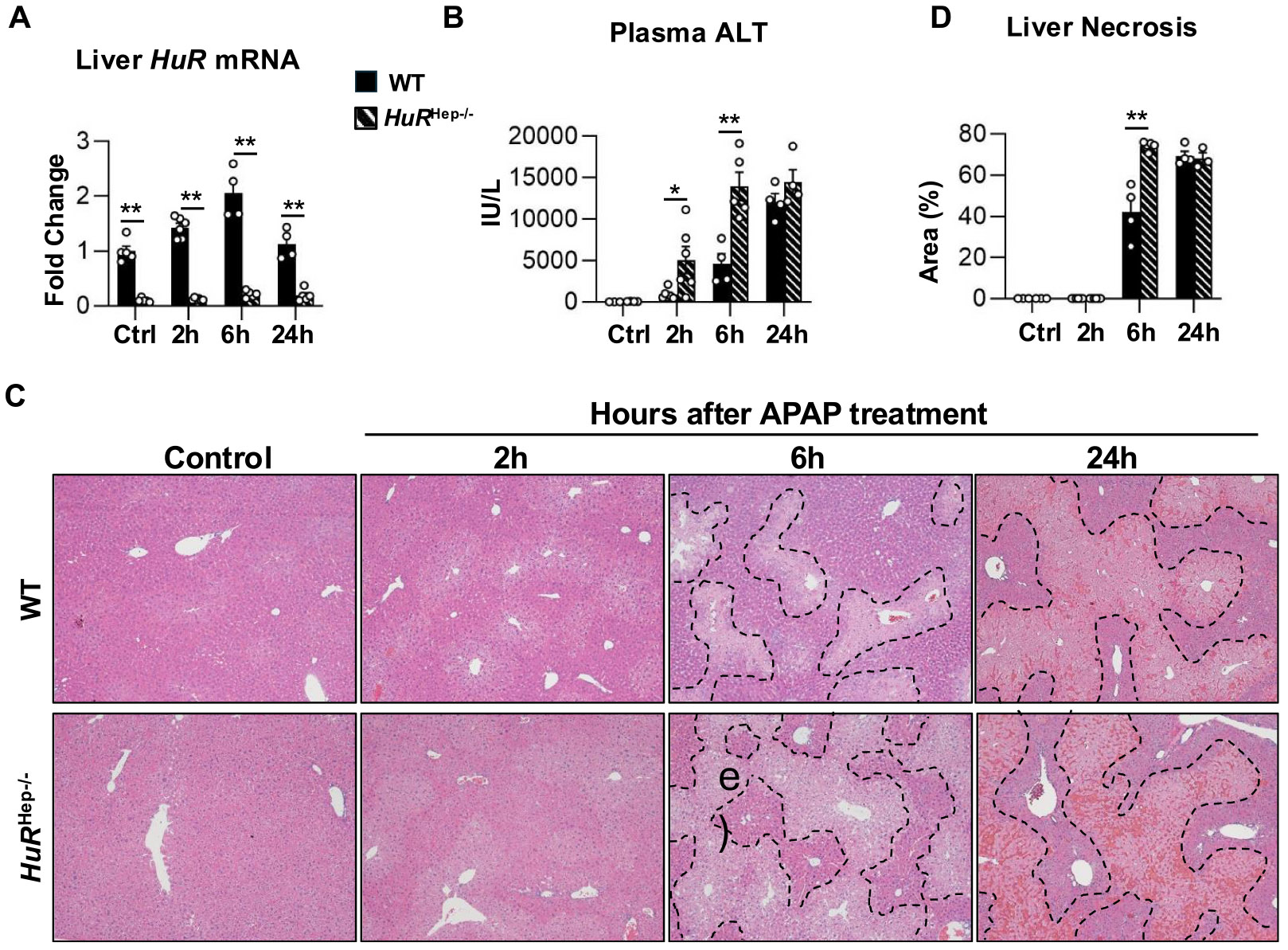
Liver injury is exacerbated in male *HuR*^Hep−/−^ mice after APAP overdose. HuR expression and liver injury were assessed at indicated time points following 200 mg/kg APAP. (A) qPCR analysis of liver HuR mRNA expression. (B) Plasma alanine aminotransferase (ALT) following APAP overdose. (C) Representative images of H&E-stained liver sections with dashed lines demarcating necrotic area. (D) Quantification of liver necrosis area outlined in C. Data are represented as mean ± SEM. In A, B, and D, each data point represents an individual mouse and n = 3–6 mice per group. Comparisons involving multiple genotypes and time points were analyzed using two-way ANOVA followed by Sidak’s multiple comparisons test. * p < 0.05, ** p < 0.01. Abbreviations: APAP, acetaminophen; H&E, hematoxylin and eosin; HuRHep−/−, hepatocyte-specific HuR knockout; WT, wild type.

We next assessed the progression of APAP-induced liver injury in WT and *HuR*^Hep−/−^ mice. Liver tissues and plasma were collected at 2, 6, and 24 h after APAP overdose. Quantitative PCR confirmed the efficient *HuR* depletion in *HuR*^Hep−/−^ livers (Fig. 2A). As expected, plasma ALT levels and hepatic necrotic area increased over time following APAP administration in both genotypes (Fig. 2B–D). Compared with WT mice, *HuR*^Hep−/−^ mice exhibited significantly higher plasma ALT levels at both 2 h (WT vs *HuR*^Hep−/−^, p = 0.04) and 6 h (WT vs *HuR*^Hep−/−^, p < 0.0001) post-overdose (Fig. 2B), along with more extensive liver necrosis at 6 h (WT vs *HuR*^Hep−/−^, p < 0.001) (Fig. 2D). In contrast, the severity of liver injury was comparative between genotypes at 24 h post overdose (Fig. 2B–D). Overall, these data indicate that hepatocyte *HuR* deficiency is associated with increased early liver injury following APAP overdose (200 mg/kg), without a detectable difference in injury severity at later time points.

### 3.3. FTIR analysis reveals distinct biochemical signatures in WT and HuR^Hep−/−^ livers before and after 2-hour APAP exposure

**Fig. 3.**
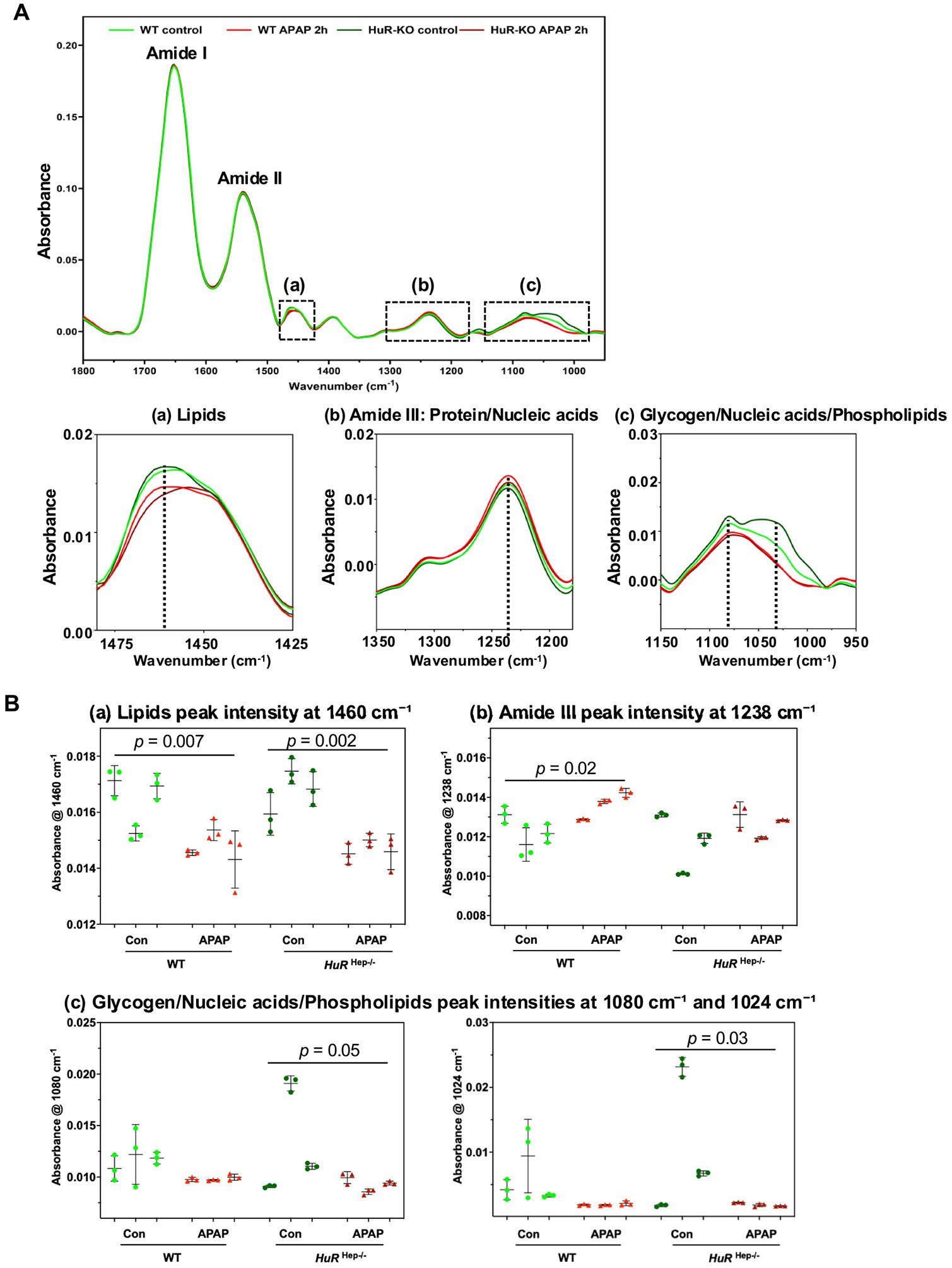
FTIR analysis reveals distinct biochemical signatures in WT and *HuR*^Hep−/−^ livers before and after 2-hour APAP exposure. Representative FTIR spectra were obtained from regions of interest (ROIs) located near the central vein. (A) FTIR spectral plots highlighting characteristic wavenumber regions corresponding to (a) lipids, (b) amide III (proteins/nucleic acids), and (c) glycogen/nucleic acids/phospholipids. (B) Average maximum intensities at (a) 1460 cm^−1^ , (b) 1238 cm^−1^ , and (c) 1080 cm^−1^ (left) and 1024 cm^−1^ (right) across treatment groups are presented in a separated scatter format. Data are represented as mean ± SEM. In A and B, n = 3 mice per group and technical replicates are grouped by individual animal to illustrate within- and between-animal variability. Animals selected for FTIR analysis were chosen based on H&E staining and serum ALT levels that most closely reflected the average injury severity of the respective group. Data were analyzed using a nested one-way ANOVA with a mixed-effects model (REML), followed by Sidak’s multiple comparisons test to calculate p value. Abbreviations: FTIR, Fourier transform infrared; APAP, acetaminophen; *HuR*^Hep−/−^, hepatocyte-specific *HuR* knockout; WT, wild type.

FTIR spectroscopy was applied to liver sections from control and 2 h APAP-treated WT and *HuR*^Hep−/−^ mice to characterize subtle biochemical differences associated with *HuR* deficiency and early APAP-induced injury. Representative FTIR spectra from three regions of interest (ROIs) located near central vein regions per sample and three animals per group were analyzed and compared (Supplementary Figure 2). The animal selected for FTIR analysis was chosen based on H&E staining and serum ALT levels that most closely reflected the average injury severity of the respective group. Spectral changes were observed across three regions: (a) 1480 – 1420 cm_−1_ , corresponding primarily to lipids; (b) 1350 – 1200 cm_−1_ , associated with the amide III band of protein and nucleic acids; and (c) 1150 – 950 cm_−1_ , reflecting contributions from glycogen, nucleic acids, and phospholipid (Fig. 3A). Changes were assessed based on peak height/intensity and peak shifts, which reflect quantity and structural alterations of these molecules. Because multiple ROIs were obtained per animal, the FTIR data exhibit a hierarchical structure (ROIs nested within animals). All FTIR datasets were analyzed using a nested one-way ANOVA with a mixed-effects model (REML). This hierarchical approach partitions variance between biological replicates (independent animals; n = 3 per group) and technical replicates (3 ROIs per animal), ensuring that statistical inference is properly conducted at the animal level.

In the lipid-associated 1480 – 1420 cm_−1_ region, both WT and *HuR*-^Hep−/−^ mice exhibited significantly reduced peak intensity at 1460 cm_−1_ following 2 h of APAP treatment compared with their respective controls (WT, p = 0.007; *HuR*^Hep−/−^, p = 0.002) (Fig. 3B). In addition, the *HuR*^Hep−/−^ APAP 2 h group displayed a band shift toward lower wavenumbers compared to other groups, suggesting APAP-induced structural alterations in lipid molecules that may be accentuated by *HuR* deficiency (Fig. 3A). Within the 1350 – 1200 cm_−1_ region, the WT APAP 2 h group exhibited a significant increase in peak intensity at 1238 cm_−1_ compared with untreated WT controls (p = 0.02), whereas this APAP-induced increase was absent in *HuR*^Hep−/−^ livers (Fig. 3B). In the 1150 – 950 cm_−1_ spectral region, only APAP-treated *HuR*^Hep−/−^ livers exhibited reduced peak intensities at 1080 cm^−1^ (p = 0.05) and 1024 cm^−1^ (p = 0.03), consistent with reduced hepatic glycogen content after APAP treatment (Fig. 3B). There findings suggest that *HuR* loss alters the early metabolic response to APAP-induced liver injury. Additionally, the *HuR*^Hep−/−^ control group exhibited greater inter-sample variability at 1080 cm_−1_ and 1024 cm_−1_ (Fig. 3B), indicating baseline metabolic heterogeneity in *HuR*-deficient livers. No significant differences between WT and *HuR*^Hep−/−^ groups were detected in the amide I (1700 – 1600 cm_−1_) or amide II (1570 – 1500 cm_−1_) regions (Fig. 3A). In summary, FTIR spectroscopy revealed significant, region-specific biochemical alterations in liver tissue as early as 2 h after APAP exposure in both WT and *HuR*^Hep−/−^ mice, with *HuR* deficiency exacerbating or modifying lipid- and glycogen-associated spectral changes during early hepatotoxic injury.

### 3.4. Altered APAP metabolite disposition in HuR^Hep−/−^ mice 2 h after APAP overdose

**Fig. 4.**
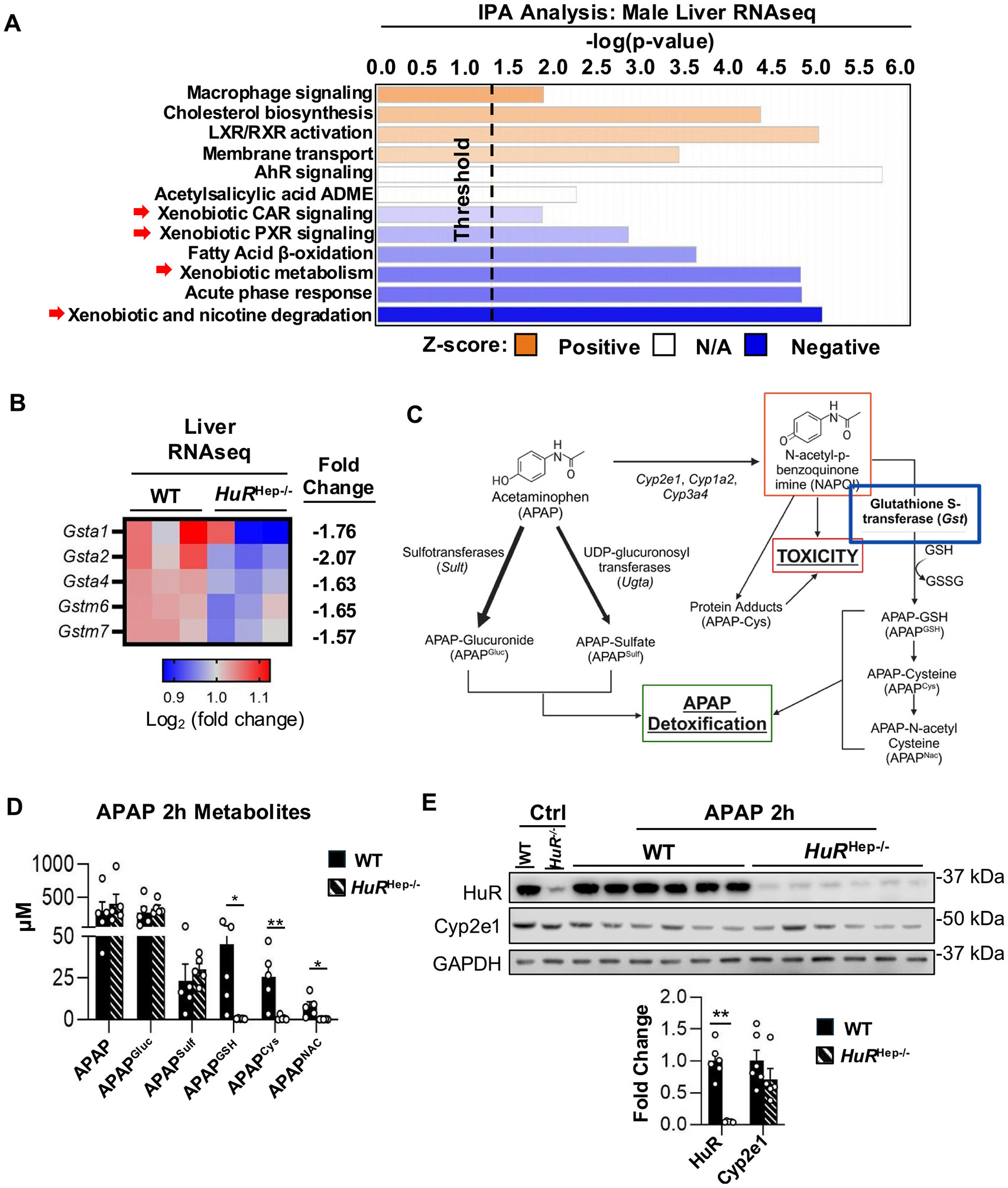
Dysregulation of APAP metabolism in *HuR*^Hep−/−^ h after APAP overdose. (A) IPA-based canonical pathway analysis of liver RNA-seq from untreated WT and *HuR*^Hep−/−^ male mouse livers (n = 3 mice per group); significantly dysregulated pathways had a −log(p-value) > 1.3; Z > 0 = pathway activation, Z < 0 = pathway inhibition. (B) Heatmap of significantly repressed glutathione S-transferases (Gst) identified in the WT vs *HuR*^Hep−/−^ RNA-seq comparison. n = 3 mice per group. (C) Schematic of hepatic APAP metabolism. (D) LC-MS/MS analysis of APAP metabolites in plasma collected 2 h following 200 mg/kg APAP treatment. (E) Western blot analysis of HuR and Cyp2e1 protein expression. Band intensity was quantified and normalized to GAPDH. Data are represented as mean ± SEM. In D and E, n = 5–6 mice per group. Each data point represents an individual mouse. Statistical differences between 2 groups were analyzed using an unpaired 2-tailed Student’s t test. * p < 0.05, ** p < 0.01. Abbreviations: RNA-seq, RNA sequencing; IPA, Ingenuity Pathway Analysis; Cyp2e1, cytochrome P450 2e1; GAPDH, glyceraldehyde-3-phosphate dehydrogenase.

Efficient early detoxification and elimination of reactive APAP metabolites are critical for limiting liver injury following APAP overdose (McGill and Jaeschke, 2013). Bulk liver RNA-seq analysis of untreated WT and *HuR*^Hep−/−^ livers revealed dysregulation of multiple metabolic pathways in *HuR*^Hep−/−^ mice, including pathways associated with xenobiotic metabolism (Fig. 4A). More specifically, several significantly repressed DEGs identified in *HuR*^Hep−/−^ livers encoded glutathione S-transferases (*Gsts*), including *Gsta1*, *Gsta2*, *Gsta4*, *Gstm6*, and *Gstm7* (Fig. 4B). These cytosolic, phase II detoxification enzymes catalyze the conjugation of glutathione (GSH) to electrophilic metabolites such as the reactive APAP metabolite NAPQI, as well as the glutathionylation of redox-sensitive proteins (Coles et al. 1988; Mazari et al. 2023). Although GSH conjugation predominates at therapeutic APAP doses, GST-mediated detoxification becomes increasingly important as hepatic GSH levels decline during overdose (Coles et al. 1988).

In both mice and humans, APAP undergoes extensive hepatic metabolism, and plasma levels of APAP and its downstream metabolites reflect hepatic metabolic capacity (Fischer et al. 1981; Prescott, 1980) (Fig. 4C). To evaluate APAP metabolism in *HuR*^Hep−/−^ mouse, APAP metabolites were quantified in plasma collected from WT and *HuR*^Hep−/−^ mice 2 h after 200 mg/kg APAP overdose. Plasma levels of unconjugated APAP and the major phase II metabolites APAP-glucuronide (APAP^Gluc^) and APAP-sulfate (APAP^Sulf^) were comparable between WT and *HuR*^Hep−/−^ mice (Fig. 4D). In contrast, consistent with reduced GSTs expression, plasma levels of downstream products of GSH conjugation to NAPQI, including APAP-GSH (APAP^GSH^), APAP-cysteine (APAP^Cys^), and APAP N-acetylcysteine (APAP^Nac^), were dramatically decreased in *HuR*^Hep−/−^ mice relative to WT controls. Specifically, at 2 h after APAP overdose, plasma from *HuR*^Hep−/−^ mice contained 87-fold (p = 0.05), 29-fold (p = 0.01), and 78-fold (p = 0.02) lower levels of APAP^GSH^, APAP^Cys^, and APAP^Nac^, respectively (Fig. 4D). Because GSH conjugation to NAPQI occurs only after phase I bioactivation of APAP (McGill and Jaeschke, 2013), expression of the primary cytochrome P450 enzyme responsible for NAPQI formation, Cyp2e1, was also examined. However, Cyp2e1 protein levels did not differ between WT and *HuR*^Hep−/−^ mice (Fig. 4E). Collectively, these findings indicate that altered APAP metabolite disposition in *HuR*-deficient mice is unlikely to result from differences in APAP bioactivation via Cyp2e1. Rather, these findings suggest impaired downstream detoxification, particularly reduced GSH conjugation, which may increase the availability of reactive NAPQI for covalent binding to hepatic proteins, a well-established contributor to APAP-induced liver injury.

### 3.5. Aberrant mitochondrial structure and function in HuR^Hep−/−^ livers before and after APAP exposure

**Fig. 5.**
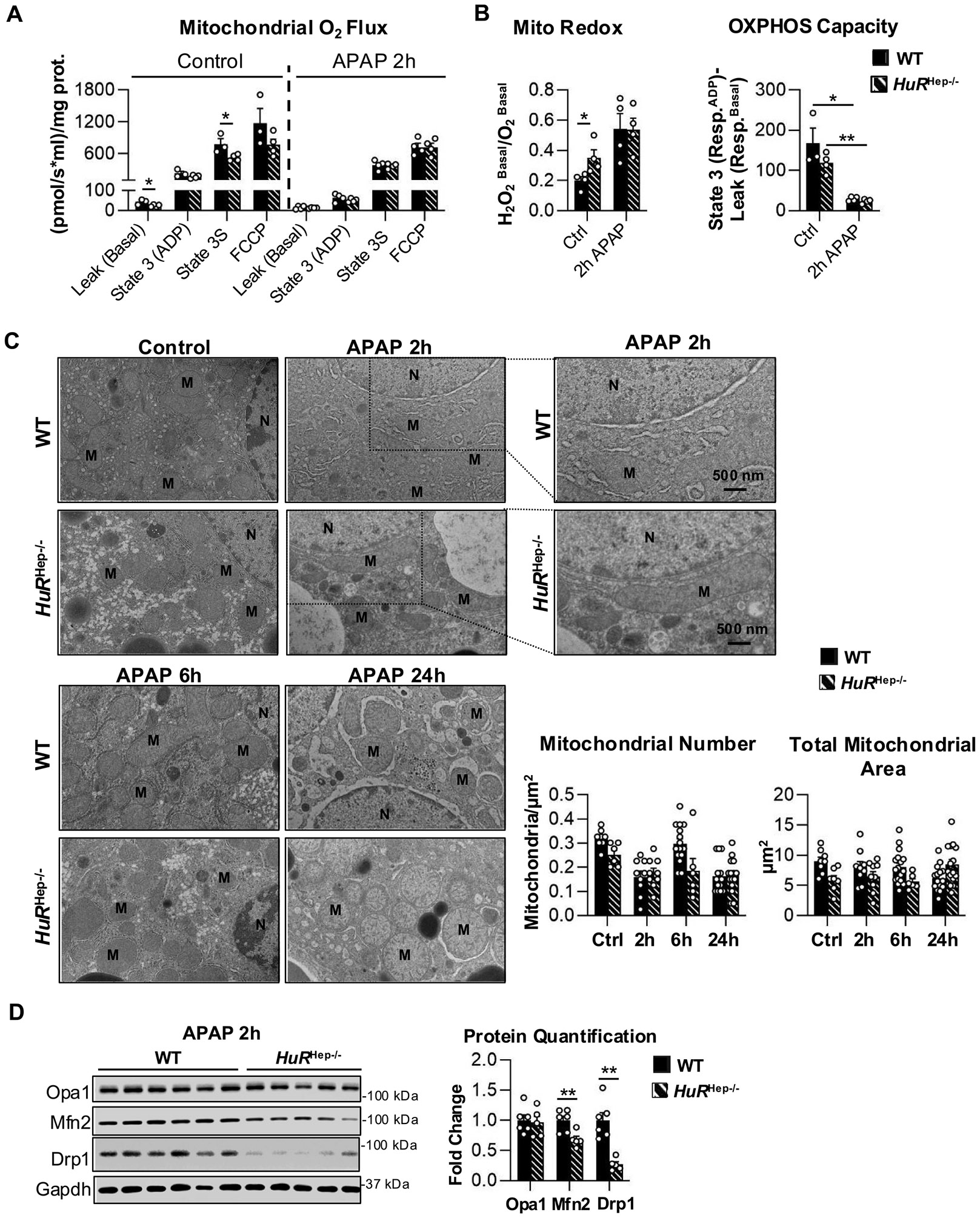
Mitochondrial respiration, morphology, and dynamics are disrupted in *HuR*^Hep−/−^ livers before and after APAP exposure. (A) Mitochondrial respiration and H_2_O_2_ production were measured using an Oroboros O2k fluorometer in mitochondria isolated from WT and *HuR*^Hep−/−^ livers under untreated (control) conditions and at 2 h after APAP treatment (2 h APAP). Oxygen consumption was assessed sequentially following the addition of malate, ADP, succinate, and FCCP. n = 3–4 mice per group. (B) Mitochondrial redox status, expressed as basal H_2_O_2_ production normalized to basal O_2_ flux, and OXPHOS capacity, calculated as State 3 respiration minus leak respiration. n = 3–4 mice per group. (C) Representative transmission electron micrographs (TEMs) of WT and *HuR*^Hep−/−^ mouse livers collected under control conditions and at indicated time points following APAP treatment. TEM analysis was performed using one mouse per genotype and time point. Mitochondrial features were quantified at the organelle level and are presented as within-animal distributions. Mitochondrial number represents the total number of mitochondria per micrograph, whereas mitochondrial area represents the total surface area occupied by mitochondria in each micrograph. (D) Western blot analysis of mitochondrial dynamics proteins Opa1, Mfn2, and Drp1 in livers collected 2 h after APAP treatment. Protein levels were normalized to GAPDH. Data are represented as mean ± SEM. n = 5–6 mice per group. Each data point represents an individual mouse. Statistical differences between 2 groups were analyzed using an unpaired 2-tailed Student’s t test. Comparisons involving multiple genotypes and time points were analyzed using two-way ANOVA followed by Sidak’s multiple comparisons test. * p < 0.05, ** p < 0.01. Abbreviations: ADP, adenine diphosphate; FCCP, carbonyl cyanide-p-(trifluoromethyoxy) phenylhydrazone; M, mitochondria; N, nucleus, Opa1, optic atrophy 1; Mfn2, mitofusion 2; Drp1, dynamin-related protein 1.

Mitochondrial dysfunction is central to the progression of APAP-induced hepatotoxicity (Ramachandran and Jaeschke 2019b). Mechanistically, APAP overdose impairs mitochondrial respiratory function in hepatocytes (Burcham and Harman, 1991), while inherent defects in the electron transport chain (ETC) function predispose mitochondria to injury (Vercellino and Sazanov, 2022). To assess mitochondrial respiration, we used an O2k fluorometer to measure respiration and H_2_O_2_ production of mitochondria isolated from untreated WT and *HuR*^Hep−/−^ male livers, as well as from WT and *HuR*^Hep−/−^ livers collected 2 h after 200 mg/kg APAP treatment. Various electron transport chain substrates (pyruvate, ADP, glutamate, malate) and the uncoupler, FCCP, were added to mitochondria within the fluorometric chamber to assess mitochondrial respiratory capacity. Overall, mitochondrial respiration increased with the addition of each substrate and reached maximal levels following FCCP addition in mitochondria isolated from both WT and *HuR*^Hep−/−^ mice (Fig. 5A). However, leak (basal) respiration and succinate-driven State 3 (State 3S) respiration were decreased significantly in mitochondria from untreated *HuR*^Hep−/−^ livers relative to WT controls (basal, p = 0.04; State 3S, p = 0.04) (Fig. 5A). In addition, mitochondrial redox status (H_2_O_2_^Basal^/O_2_^Basal^) was elevated in mitochondria isolated from untreated *HuR*^Hep−/−^ livers relative to WT (p = 0.03), although OXPHOS capacity (Resp.^ADP^-Resp.^Basal^) did not differ between genotypes (Fig. 5B). Following APAP treatment, mitochondrial respiration was impaired in both WT and *HuR*^Hep−/−^ mice, as indicated by markedly reduced OXPHOS capacity and increased redox potential in mitochondria isolated 2 h after APAP overdose (Fig. 5B). Because proper assembly and function of the electron transport chain (ETC) are essential for mitochondrial respiration and limiting oxidant stress (Vercellino and Sazanov, 2022), we next assessed ETC protein expression. Western blot analysis revealed no differences in the expression of representative proteins from ETC complexes I-V between WT and *HuR*^Hep−/−^ livers under either control or APAP-treated conditions (Supplementary Figure 3 A). Collectively, these findings indicate that impaired mitochondrial respiration in untreated *HuR*^Hep−/−^livers occurs independently of changes in ETC protein abundance and may predispose mitochondria to increased oxidant stress.

Defects in mitochondrial structure are closely linked to mitochondrial dysfunction under conditions of cellular stress (Eisner et al. 2018). To assess mitochondrial morphology, transmission electron microscopy (TEM) was used to examine hepatocyte mitochondria from untreated livers and from livers collected 2, 6, and 24 h following APAP overdose. For each genotype and time point, one mouse was analyzed, and multiple hepatocytes and mitochondria were quantified per animal. The animal selected for TEM analysis was chosen based on H&E staining and serum ALT level that most closely reflected the average injury severity of the respective group. Because TEM analysis was performed on a single animal per group at each time point and mitochondrial features were quantified at the organelle level, these data are presented as within-animal observations and should be considered descriptive. Additional biological replicates will be required for statistical validation. Overall, mitochondrial number and morphology in hepatocytes appeared to change dynamically in response to APAP exposure and hepatocyte-specific *HuR* deficiency (Fig. 5C). In untreated conditions, hepatocytes from *HuR*^Hep−/−^ livers contained fewer mitochondria and reduced total mitochondrial area compared with WT controls (Fig. 5C). Despite this reduction, mitochondria in *HuR*^Hep−/−^ hepatocytes displayed a more circular morphology, as quantified by the circularity index (4π × area/perimeter^2^; ImageJ) (Fig. 5C and Supplementary Figure 3B). At 2 h following APAP overdose, mitochondrial number appeared decreased in both WT and *HuR*^Hep−/−^ hepatocytes relative to untreated controls; however, mitochondria at this time point exhibited a more elongated morphology (Fig. 5C). Importantly, extremely large and elongated mitochondria were observed specifically in *HuR*^Hep−/−^ livers at 2 h post-APAP exposure (Fig. 5C, insert). By 6 h post-overdose, mitochondrial number appeared to increase in WT hepatocytes relative to the 2-hour time point, whereas this increase was not observed in *HuR*^Hep−/−^ livers (Fig. 5C). Correspondingly, *HuR*^Hep−/−^ hepatocytes exhibited reduced mitochondrial area compared with WT at this time point (Fig. 5C). At 24 h, mitochondrial circularity and surface area in WT hepatocytes appeared comparable to untreated controls. In contrast, *HuR*^Hep−/−^ hepatocytes continued to exhibit higher mitochondrial circularity and surface area relative to WT (Fig. 5C and Supplementary Figure 3B). Taken together, these descriptive TEM observations suggest that both APAP exposure and hepatocyte-specific *HuR* deficiency are associated with alterations in mitochondrial morphology. In particular, the dynamic changes in mitochondrial number observed in WT livers, characterized by an early decrease at 2 h, partial recovery at 6 h, and subsequent decline at 24 h, appeared attenuated in *HuR*^Hep−/−^ livers, suggesting impaired mitochondrial remodeling in response to APAP-induced stress.

Canonical mitochondrial fusion and fission proteins play essential roles in large-scale remodeling of mitochondrial structure and function (Eisner et al. 2018). Accordingly, we next assessed the expression of key mitochondrial fusion and fission proteins 2 h after APAP overdose, a time point associated with pronounced morphological alterations. At this time point, *HuR*^Hep−/−^ livers exhibited a 1.5-fold reduction in the outer mitochondrial membrane fusion protein mitofusin 2 (Mfn2) and a 2.4-fold reduction in the cytoplasmic mitochondrial fission protein dynamin-related protein 1 (Drp1) compared with WT livers (Mfn2, p = 0.01; Drp1, p = 0.007) (Fig. 5D). In contrast, levels of optic atrophy 1 (Opa1), an inner mitochondrial membrane fusion protein, were unchanged between WT and *HuR*^Hep−/−^ livers (Fig. 5D). Collectively, the coordinated reduction in Mfn2 and Drp1 expression is consistent with the aberrant mitochondrial morphology observed in *HuR*^Hep−/−^ hepatocytes and provides a mechanistic link between disrupted mitochondrial dynamics and the impaired respiratory capacity observed in *HuR*-deficient livers. Together, these findings indicate that hepatocyte *HuR* deficiency compromises mitochondrial dynamics, thereby exacerbating both structural remodeling and functional mitochondrial deficits under basal conditions and following APAP-induced liver injury.

### 3.6. Increased mitochondrial protein release, oxidant stress, and cell death in HuR^Hep−/−^ livers 2 h after APAP overdose

**Fig. 6.**
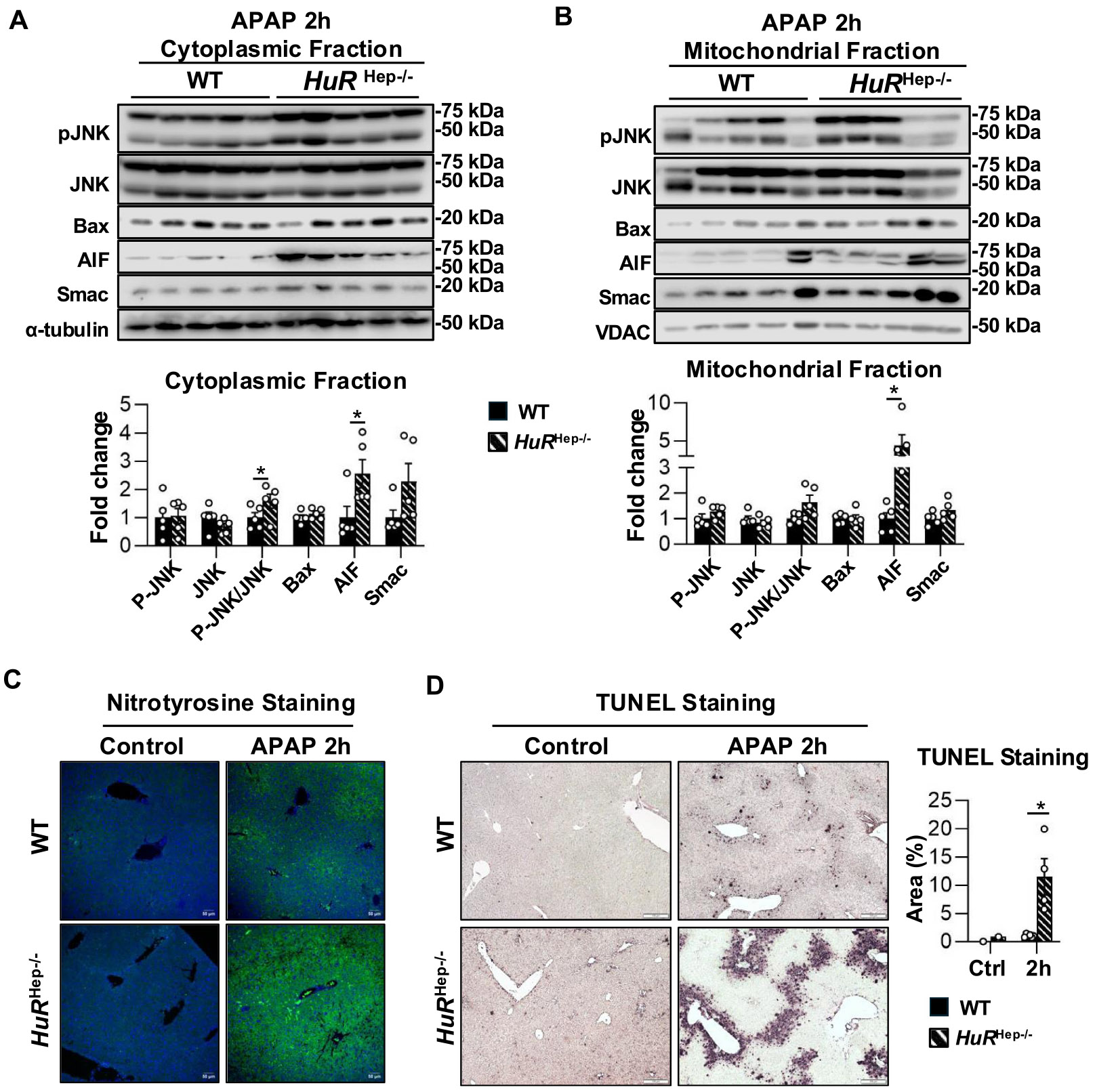
Increased mitochondrial oxidant stress and mitochondrial protein release in APAP-treated *HuR*^Hep−/−^ h after APAP overdose. (A-B) Western blot analysis of proteins isolated from cytoplasmic (A) and mitochondrial (B) liver fractions collected 2 h after APAP overdose. Protein levels were quantified densito-metrically and normalized to VDAC (mitochondrial) or α-tubulin (cytosolic). n = 5 mice per group. (C) Representative immunofluorescence images of nitrotyrosine staining in liver sections collected 2 h after APAP overdose. (D) Representative images of TUNEL staining in liver sections collected 2 h after APAP overdose, positive staining area quantified using Fiji (image J). Data are represented as mean ± SEM, n = 4 mice per group for C and D. Statistical differences between 2 groups were analyzed using an unpaired 2-tailed Student’s t test. Comparisons involving multiple genotypes and time points were analyzed using two-way ANOVA followed by Sidak’s multiple comparisons test. * p < 0.05, ** p < 0.01. Abbreviations: pJNK, phosphorylated c-Jun N terminal kinase; AIF, apoptosis-inducing factor; VDAC, voltage-dependent anion-selective channel 1; TUNEL, Terminal deoxynucleotidyl transferase dUTP nick end labeling.

Defects in mitochondrial respiration and morphology contribute to APAP-induced hepatotoxicity by promoting mitochondrial oxidant stress and release of mitochondrial proteins into the cytosol (Picard et al. 2013). Accordingly, markers of mitochondrial oxidant stress and mitochondrial dysfunction were assessed in WT and *HuR*^Hep−/−^ mouse livers at 2 h following APAP overdose (Fig. 6). Phosphorylation and mitochondrial translocation of c-JUN N-terminal kinase (pJNK) are established indicators of JNK activation and increased mitochondrial ROS production (Ramachandran and Jaeschke 2019a). At 2 h post-overdose, the pJNK/JNK ratio was increased 1.6-fold in the cytoplasmic fraction of *HuR*^Hep−/−^ male mouse livers relative to WT (p = 0.04) (Fig. 6A). In contrast, mitochondrial translocation of pJNK and total JNK did not differ between WT and *HuR*^Hep−/−^ mouse livers at this time point (Fig. 6B), suggesting that JNK activation was unlikely to be a major contributor to the heightened liver injury observed in *HuR*^Hep−/−^ mice at this time point.

To more directly assess mitochondrial oxidant stress, immunofluorescence staining for nitrotyrosine protein adducts was performed (Fig. 6C). Following APAP overdose, nitrotyrosine adduct formation is largely restricted to mitochondria damaged by ROS, making nitrotyrosine adducts a relatively specific marker of mitochondrial oxidant stress (Cover et al. 2005). Consistent with enhanced mitochondrial oxidative stress, immunofluorescence staining revealed visibly higher levels of nitrotyrosine adducts in *HuR*^Hep−/−^ male mouse livers compared with WT at 2 h post-overdose (Fig. 6C). In parallel, levels of the mitochondrial intermembrane space protein apoptosis-inducing factor (AIF), a marker of severe mitochondrial dysfunction (Bajt et al. 2006; Masubuchi et al. 2005), were markedly increased in both mitochondrial and cytoplasmic fractions of *HuR*^Hep−/−^ male mouse livers relative to WT (Fig. 6A and B). Specifically, AIF levels were increased 2.6-fold (p = 0.04) in cytoplasmic mitochondrial fractions and 4.4-fold (p = 0.03) in mitochondrial fractions of *HuR*^Hep−/−^ livers at 2 h following APAP overdose. Following release into the cytoplasm, AIF translocates to the nucleus, where it functions as an endonuclease that cleaves DNA and promotes hepatocyte necrosis (Bajt et al. 2006). Consistent with increased mitochondrial AIF release and indicative of DNA damage, terminal deoxynucleotidyl transferase dUTP nick-end labeling (TUNEL) staining was increased approximately 13-fold (p = 0.03) in *HuR*^Hep−/−^ mouse livers compared with WT at 2 h post-overdose (Fig. 6D). Taken together, these findings suggest that enhanced mitochondrial dysfunction and oxidative stress are key drivers of early hepatocyte necrosis in *HuR*^Hep−/−^ male mouse livers following APAP overdose.

### 3.7. Persistent mitochondrial dysfunction, enhanced APAP-protein adduct accumulation, and impaired glutathione recovery exacerbate hepatotoxicity in HuR-deficient livers at 6 h after APAP overdose

**Fig. 7.**
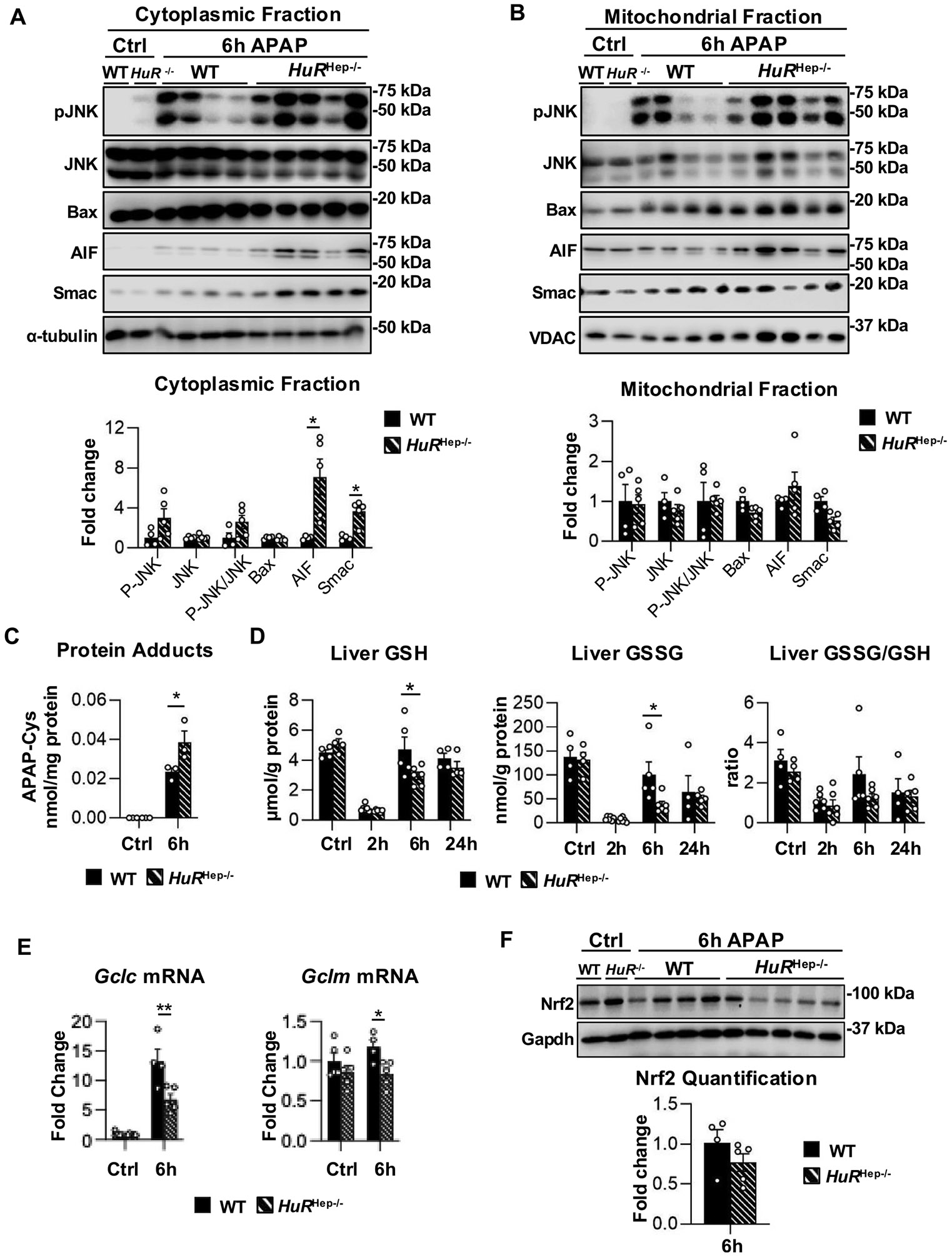
Persistent mitochondrial dysfunction, enhanced APAP-protein adduct accumulation, and impaired glutathione recovery in *HuR*^Hep−/−^ h after APAP overdose. (A-B) Western blot analysis of proteins isolated from cytoplasmic (A) and mitochondrial (B) liver fractions collected 6 h after APAP overdose. Protein levels were quantified densitometrically and normalized to α-tubulin (cytosolic) or VDAC (mitochondrial). (C) High performance liquid chromatography (HPLC) quantification of APAP-protein adducts (APAP-Cys) in WT and *HuR*^Hep−/−^ livers at 6 h after APAP overdose. (D) Hepatic glutathione (GSH) and oxidized glutathione (GSSG) levels measured using a modified Tietze method at indicated time points following APAP overdose. (E) qPCR analysis of liver *Gclc* and *Gclm* mRNA expression over time following APAP overdose. (F) Western blot analysis of Nrf2 in livers collected 6 h after APAP treatment. Protein levels were normalized to Gapdh. Data are represented as mean ± SEM. In A-F, n = 4–5 mice per group. Each data point represents an individual mouse. In E, the amplification results were normalized to the expression of the housekeeping gene hypoxanthine guanine phosphoribosyl transferase 1 (*Hprt1*) and presented as fold change relative to the control group. Comparisons involving multiple genotypes and time points were analyzed using two-way ANOVA followed by Sidak’s multiple comparisons test. * p < 0.05, ** p < 0.01. Abbreviations: pJNK, phosphorylated c-Jun N-terminal kinase; AIF, apoptosis-inducing factor; VDAC, voltage-dependent anion-selective channel 1; GSH; glutathione; Gclc, glutamate cysteine ligase catalytic subunit; Gclm, glutamate cysteine ligase modifier subunit.

More severe liver injury persisted in *HuR*^Hep−/−^ male mouse livers at 6 h following APAP overdose (Fig. 2). Accordingly, canonical markers of mitochondrial dysfunction and APAP-induced hepatotoxicity were assessed in WT and *HuR*^Hep−/−^ livers at this later time point. As observed at 2 h, cytoplasmic JNK activation was modestly increased in *HuR*^Hep−/−^ male mouse livers but did not reach statistical significance, and overall JNK activation and mitochondrial translocation were comparable between WT and *HuR*^Hep−/−^ livers at 6 h (Fig. 7A and B). In contrast, cytoplasmic levels of the mitochondrial intermembrane proteins AIF and Smac were robustly increased in *HuR*^Hep−/−^ male mouse livers compared to WT (Fig. 7A). Specifically, AIF and Smac levels were increased 7.1-fold (p = 0.038) and 3.6-fold (p = 0.005), respectively, in cytoplasmic fractions of *HuR*^Hep−/−^ livers at 6 h post-overdose (Fig. 7A).

Altered APAP metabolite disposition in HuR-deficient mice (Fig. 4D) may impair downstream detoxification, thereby increasing the availability of reactive NAPQI for covalent binding to hepatic proteins and formation of APAP-Cys protein adducts, a well-established contributor to APAP-induced liver injury (Jaeschke and Ramachandran, 2024). Consistent with this, APAP-Cys adduct levels were increased 1.65-fold (p = 0.048) in HuR^Hep−/−^ male mouse livers compared with WT at 6 h post-overdose (Fig. 7C). In parallel, hepatic levels of GSH and glutathione disulfide (GSSG), the oxidized form of the GSH, were reduced by 1.6-fold (p = 0.05) and 2.16-fold (p = 0.03), respectively, in HuR^Hep−/−^ male mouse livers relative to WT at the 6-hour time point (Fig. 7D). Glutamate cysteine ligase (Gcl), the rate-limiting enzyme in GSH synthesis, consists of a catalytic (Gclc) and a modifier (Gclm) subunits (Lu, 2013). Consistently, Gclc and Gclm mRNA levels were significantly reduced by approximately 1.82-fold (p = 0.0009) and 1.4-fold (p = 0.05), respectively, in HuR^Hep−/−^ livers compared with WT livers at 6 h (Fig. 7E), providing a mechanistic basis for the delayed recovery of hepatic GSH levels observed at this time point (Fig. 7D). Nuclear factor erythroid 2-related factor 2 (Nrf2) is a key transcriptional regulator of antioxidant-response element genes, including Gclc and Gclm. A trend toward lower Nrf2 protein levels was observed in HuR^Hep−/−^ male livers compared with WT at the 6-hour time point (Fig. 7F), consistent with the reduced induction of Gclc and Gclm. Taken together, these findings suggest that increased accumulation of APAP-Cys protein adducts, accompanied by impaired GSH recovery, potentially due to reduced induction of Nrf2, Gclc, and Gclm, contributes to the persistent liver injury observed in HuR^Hep−/−^ male mice at 6 h following APAP overdose. Additional studies, including HuR rescue experiments, will be necessary to fully establish the causal mechanisms involved.

### 3.8. Impaired hepatocyte proliferation in both WT and HuR-deficient livers at 24 h after APAP overdose

**Fig. 8.**
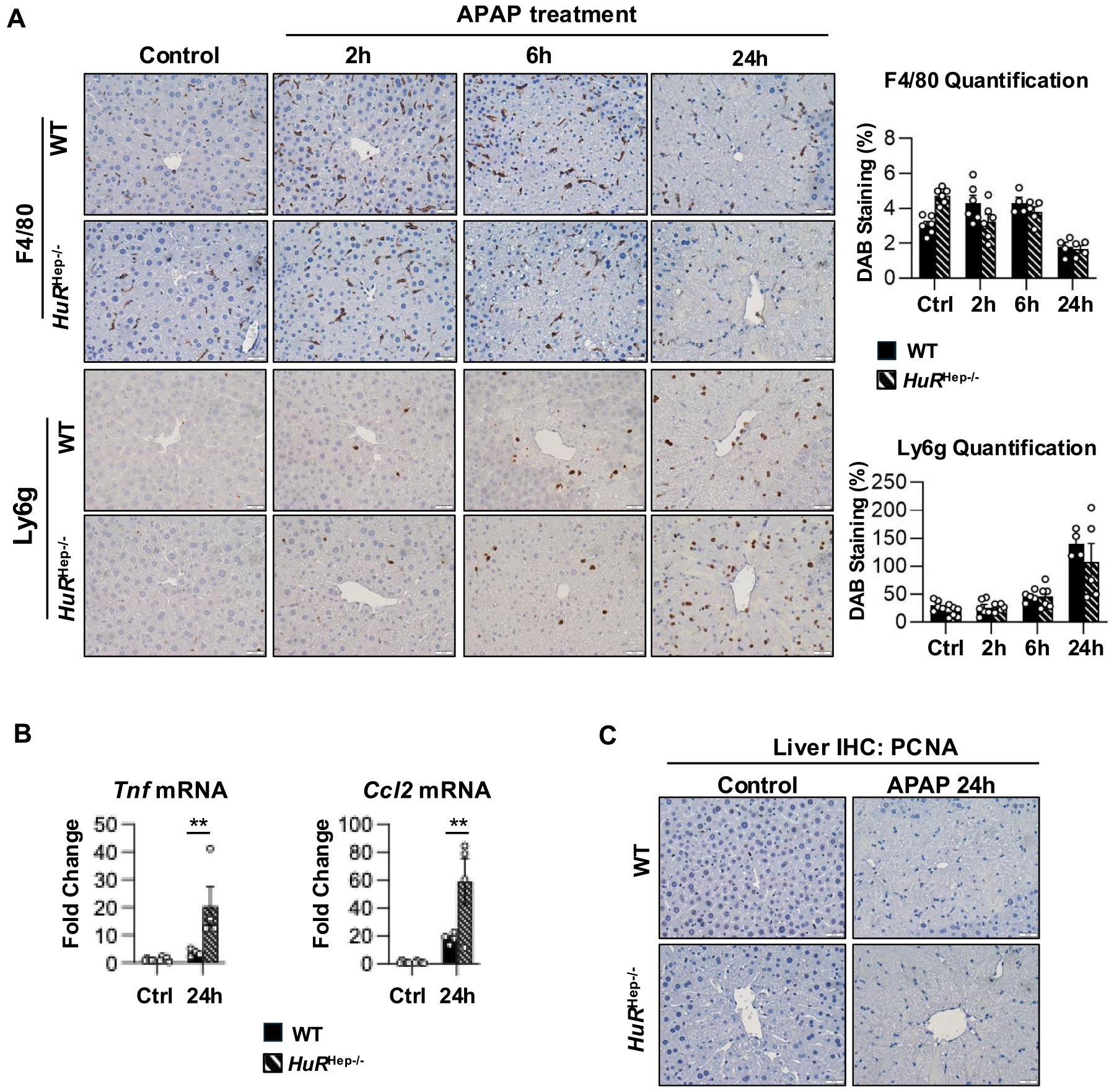
Loss of HuR enhances pro-inflammatory gene expression following APAP-overdose. (A) Representative IHC images of liver sections stained for F4/80 (top) and Ly6g (bottom). Percent positive staining area and neutrophil number were quantified using Image J. (B) qPCR analysis of hepatic *Ccl2* and *Tnfa* mRNA expression. (C) Representative IHC images of liver sections stained for PCNA. Data are represented as mean ± SEM. In A-C, n = 3–5 mice per group. Each data point represents an individual mouse. The amplification results were normalized to the expression of the housekeeping gene hypoxanthine guanine phosphoribosyl transferase 1 (*Hprt1*) and presented as fold change relative to the control group. Comparisons involving multiple genotypes and time points were analyzed using twoway ANOVA followed by Sidak’s multiple comparisons test. * p < 0.05, ** p < 0.01. Abbreviations: Ly6g, lymphocyte antigen 6; DAB, 3,3’-diaminobenzidine; Ccl2, C-C motif chemokine ligand 2; Tnfa, tumor necrosis factor alpha.

Sterile inflammation – mainly driven by the activity of macrophages and neutrophils – plays a crucial role in resolving liver injury following APAP overdose (Jaeschke and Ramachandran, 2020; Rodrigues et al. 2023). Immunohistochemistry (IHC) staining for the macrophage marker, F4/80, and the neutrophil marker, lymphocyte antigen 6 family member G (Ly6g), revealed similar immune cell abundance in WT and *HuR*^Hep−/−^ livers following APAP overdose (Fig. 8A). However, qPCR analysis revealed significantly increased expression of pro-inflammatory cytokines and chemokines, including tumor necrosis factor α (*Tnfa*; p = 0.005) and C-C motif chemokine ligand 2 (*Ccl2*; p = 0.0048), in *HuR*^Hep−/−^ mouse livers compared with WT livers at 24 h following APAP overdose (Fig. 8B). These findings suggest that *HuR* loss promotes a more pro-inflammatory environment compared to WT during APAP overdose-induced hepatotoxicity.

Hepatocytes proliferation is critical for liver regeneration after APAP overdose (Bhushan and Apte, 2019). To assess hepatocyte proliferation, IHC staining for proliferating cell nuclear antigen (PCNA) was performed on liver tissues from WT and *HuR*^Hep−/−^ male mice on a C57BL/6 N background. Although liver regeneration is less well characterized in the C57BL/6 N substrain, studies in C57BL/6 J mice have reported the increase of PCNA-positive hepatocytes at 24 h following a moderate APAP dose (300 mg/kg), whereas a severe dose (600 mg/kg) delays PCNA induction in male livers (Bhushan et al. 2014). In the present model, PCNA-positive hepatocytes were not detected in either WT or *HuR*^Hep−/−^ male mouse livers at 24 h following a 200 mg/kg APAP overdose compared with untreated controls (Fig. 8C). Given that C57BL/6 N mice are significantly more susceptible to APAP-induced hepatotoxicity than C57BL/6 J mice (Duan et al. 2016), these data suggest that 200 mg/kg APAP induces a severe injury in C57BL/6 N male mice, sufficient to delay the onset of liver regeneration. Taken together, these findings indicate that liver regeneration was delayed in both WT and *HuR*^Hep−/−^ male mice at 24 h following a 200 mg/kg APAP overdose.

### 3.9. Validation of HuR targets by HuR modulation and RNP-IP

**Fig. 9.**
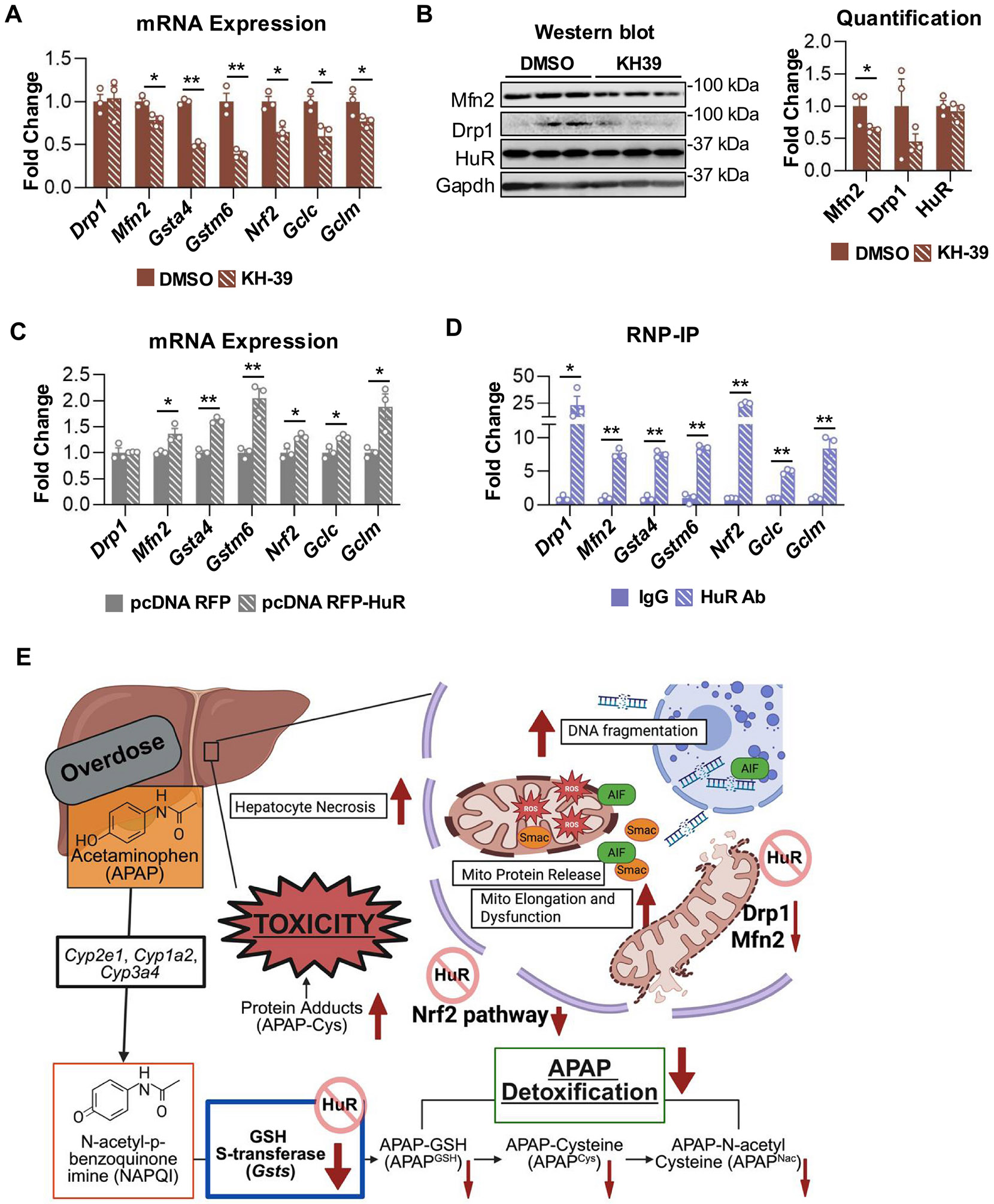
Validation of HuR target genes involved in mitochondrial dynamics, detoxification, and antioxidant responses. (A-B) Hepa1-6 cells were treated with the HuR inhibitor KH-39 (10 μM) for 48 h, with DMSO as the vehicle control. For each condition, cells were plated and treated in triplicate. mRNA expression was analyzed by qPCR (A), and protein levels were assessed by Western blot (B). Data are presented as mean ± SEM (n = 3 per group). (C) Hepa1-6 cells were transfected with pcDNA RFP-HuR expression plasmid for 24 h, with pcDNA RFP used as the vector control. Cells were treated in triplicate, and mRNA expression was analyzed by qPCR. Data are presented as mean ± SEM (n = 3 per group). (D) Ribonucleoprotein immunoprecipitation (RNP-IP) was performed in Hepa1-6 cells using anti-HuR or isotype IgG control antibodies, followed by qPCR analysis. Enrichment of target transcripts in HuR immunoprecipitates relative to IgG controls was calculated after normalization to the corresponding input samples. Data are presented as mean ± SEM, n = 3 technical replicates per group for qPCR. Statistical differences between two groups were analyzed using an unpaired two-tailed Student’s *t*-test. RNA-IP experiments were independently repeated twice using cells from separate culture dishes to confirm reproducible HuR binding; one representative experiment is shown in (D), and the second set is presented in Supplemental Fig 4B. (E) Schematic depicting how loss of hepatocyte HuR amplifies APAP-induced liver injury through coordinated defects in xenobiotic metabolism and mitochondrial homeostasis. HuR deficiency reduces glutathione S-transferases (Gsts) expression and disrupts APAP detoxification, leading to increased NAPQI burden and enhanced APAP-protein adduct formation. Concomitantly, impaired induction of glutathione synthesis delays GSH recovery, further limiting detoxification capacity. HuR loss also dysregulates mitochondrial dynamics by reducing Drp1 and Mfn2 expression, resulting in abnormal mitochondrial morphology, compromised respiratory function, and increased mitochondrial membrane permeabilization. These defects promote mitochondrial protein release and nuclear DNA fragmentation, thereby intensifying hepatocyte necrosis and overall liver injury in *HuR*^Hep−/−^ male mice. Abbreviations: NAPQI, N-acetyl-p-benzoquinone imine; Gst, glutathione S-transferase; GSH, glutathione; Mfn2, mitofusion 2; Drp1, dynamin-related protein 1; RNP-IP, Ribonucleoprotein immunoprecipitation.

To investigate the mechanisms by which HuR regulates detoxification and mitochondrial integrity, we queried the oRNAment database and identified multiple putative HuR recognition motifs within 3′-un-translated regions of mRNAs encoding mitochondrial dynamics proteins (Drp1, Mfn2), detoxification enzymes (Gsta4, Gstm6), and antioxidant regulators (Nrf2, Gclc, Gclm). To determine whether HuR modulates the expression of these genes, Hepa 1–6 cells were treated with a specific HuR inhibitor KH-39 or transfected with an RFP-HuR expression plasmid. KH-39 is a recently developed, potent, and selective HuR inhibitor from Dr. Liang Xu’s group that binds the HuR RNA-binding pocket and effectively disrupts HuR-ARE interactions (Liu et al. 2025; Wu et al. 2023). Treatment with 10 μM KH-39 significantly reduced mRNA expression of Mfn2 (1.3-fold decrease; p = 0.02), Gsta4 (1.99-fold decrease; p < 0.001), Gstm6 (2.52-fold decrease; p = 0.004), Nrf2 (1.54-fold decrease; p = 0.01), Gclc (1.66-fold decrease; p = 0.03), and Gclm (1.3-fold decrease; p = 0.05) compared with DMSO controls (Fig. 9A). Interestingly, although Drp1 mRNAs were not significantly altered, Drp1 protein levels showed a decreasing trend following KH-39 treatment (Fig. 9B). Consistent with previous report, inhibition of HuR by small-molecule inhibitors, can reduce protein expression of target genes without proportionally decreasing mRNA levels (Wu et al. 2020). This discrepancy likely reflects the dual, yet separable, roles of HuR in promoting mRNA stabilization and translation. When HuR binding is disrupted by inhibitor, ribosome recruitment and translation efficiency are impaired, leading to reduced protein synthesis even in the absence of immediate mRNA degradation.

In contrast, HuR overexpression significantly increased mRNA levels of Mfn2 (1.36-fold; p = 0.02), Gsta4 (1.62-fold; p < 0.001), Gstm6 (2.04-fold; p = 0.007), Nrf2 (1.32-fold; p = 0.02), Gclc (1.31-fold; p = 0.01), and Gclm (1.89-fold; p = 0.03) relative to vector controls (Fig. 9C), with HuR overexpression confirmed by Western blot (Supplementary Figure 4A). Importantly, RNP-IP demonstrated significant enrichment of HuR-bound mRNAs compared with IgG controls, including Drp1 (23.22-fold; p = 0.05), Mfn2 (7.68-fold; p = 0.0001), Gsta4 (7.56-fold; p < 0.0001), Gstm6 (8.45-fold; p = 0.0001), Nrf2 (24.74-fold; p < 0.0001), Gclc (5-fold; p < 0.0001), and Gclm (8.45-fold; p = 0.006) (Fig. 9D). RNA-IP experiments were independently repeated twice using cells from separate dishes to confirm reproducibility (Fig. 9D and Supplementary Figure 4B). Together, these findings demonstrate that HuR directly binds to and regulates mRNAs involved in mitochondrial dynamics, detoxification, and antioxidant responses, supporting its role in maintaining mitochondrial integrity and cellular detoxification capacity.

## 4. Discussion

HuR is a ubiquitously expressed RNA-binding protein that regulates cellular survival and homeostasis by modulating the stability and processing of numerous target transcripts (Eppler et al. 2025; Srikantan and Gorospe 2012). The present study comprehensively examined the role of hepatocyte HuR in APAP-induced hepatotoxicity across the metabolism/early injury (2 h), injury (6 h), and early recovery (24 h) phases following APAP overdose. After APAP exposure, increased HuR mRNA expression, HuR protein cleavage, and the appearance of higher-molecular weight HuR species closely tracked with the progression of liver injury. During the metabolism and injury phases, hepatocyte-specific *HuR* deficiency exacerbated liver injury. Specifically, enhanced liver injury during the early metabolism phase was associated with altered oxidative APAP metabolite disposition, increased nitrosative stress, and mitochondrial dysfunction. At 6 h post-overdose, more severe liver injury in *HuR*^Hep−/−^ mice was accompanied by increased APAP-protein adduct formation, delayed GSH recovery, and elevated mitochondrial protein release. Finally, although impaired hepatocyte proliferation at 24 h in both WT and *HuR*^Hep−/−^ mice reflected the overall severity of injury at this dose, *HuR*^Hep−/−^ livers exhibited distinct increases in pro-inflammatory cytokine expression. Mechanistically, RNP-IP revealed direct binding of HuR to mRNAs encoding mitochondrial dynamics proteins (Drp1, Mfn2), detoxification enzymes (Gsta4, Gstm6), and antioxidant regulators (Nrf2, Gclc, Gclm). Collectively, these findings identify HuR as a critical regulator of detoxification and mitochondrial integrity during APAP-induced hepatotoxicity (Fig. 9E).

An important finding of this study is that induction of hepatic HuR expression, together with the appearance of higher-molecular-weight HuR species and HuR cleavage products, closely parallels the time course of liver injury following APAP overdose. Cellular stress, including oxidative stress, has been shown to induce both HuR expression and HuR cleavage (Ashour et al. 2024; Banos-Jaime et al. 2024; Mazroui et al. 2008; Talwar et al. 2011; von Roretz and Gallouzi, 2010; von Roretz et al. 2013). In addition, HuR can form dimers and higher-order complexes under oxidative stress to facilitate the cellular stress response, primarily by stabilizing pro-survival mRNAs. For instance, oxidative stress can lead to HuR homodimerization at the RRM1 and RRM1/2 domains through a disulfide bond at cysteine 13, contributing to redox-dependent regulation of HuR activity under oxidative stress (Benoit et al. 2010). RRM3 dimerization through tryptophan 261 enhance HuR binding to ARE-containing mRNAs (Pabis et al. 2019). Beyond dimers, Hu/ELAV proteins can form higher-order aggregates or multimers (Soller and White, 2005; Toba and White, 2008). These multimers/aggregates are associated with promoting pro-survival mRNA expression under intense stress conditions. In addition, oxidative stress activates check-point kinase 2 (Chk2), which phosphorylates HuR (e.g., Ser100, Thr118), influencing its binding affinity to target mRNAs (Abdelmohsen et al. 2007). Methylation of HuR at Arg217 by CARM1 in response to stress enhances its ability to stabilize target mRNAs (Li et al. 2002). These mechanisms are particularly relevant in the context of APAP-induced hepatotoxicity, which is characterized by severe mitochondrial oxidative stress, reactive nitrogen species formation, and covalent protein modifications by the reactive metabolite NAPQI. Such conditions are known to promote protein crosslinking, disulfide bond formation, and stress-induced protein oligomerization. Therefore, the appearance of the HuR-HMW species during APAP-induced liver injury may reflect oxidative stress-associated HuR dimerization, oligomerization, post-translational modifications, or association with HuR-containing protein complexes. The precise molecular identity of this HuR-HMW species remains to be determined. Future studies could address this by comparing reducing versus non-reducing SDS-PAGE to assess potential disulfide-linked HuR dimers, performing immunoprecipitation followed by mass spectrometry to identify HuR-containing complexes or post-translational modifications, or using mutational analysis of cysteine or tryptophan residues involved in HuR dimerization to evaluate the contribution of redox-dependent HuR dimerization.

HuR cleavage has been shown to promote apoptotic cell death in HeLa cells under lethal stress (Mazroui et al. 2008), while also being required for muscle cell differentiation through stabilization of differentiation-related mRNAs (Beauchamp et al. 2010). In the present study, while the underlying mechanisms and functional consequences of HuR cleavage in APAP-injured livers remain unclear, the strong correlation between HuR cleavage and serum ALT levels suggests a close association with hepatocellular injury severity. Furthermore, the presence of HuR cleavage products in the absence of caspase-3 activation indicates that HuR may undergo caspase-independent cleavage during APAP-induced hepatotoxicity, consistent with the predominance of necrotic cell death in this model. Further studies are needed to define the molecular pathways governing HuR cleavage and modification during APAP-induced liver injury.

Another important finding of this study is the identification of HuR as a regulator of APAP metabolism, with Gsta4 and Gstm6 identified as direct HuR targets. Previous studies have identified a metabolism-associated function for HuR in maintaining liver homeostasis (Eppler et al. 2025). Liver RNP-IP combined with bulk RNA sequencing revealed dysregulation of multiple metabolic pathways in *HuR*-deficient mouse livers and direct HuR binding to transcripts encoding key enzymes involved in endobiotic and xenobiotic metabolism (Subramanian et al. 2022). Consistent with these findings, our study revealed marked dys-regulation of GSH-dependent APAP metabolism in *HuR*^Hep−/−^ mouse livers. Specifically, plasma levels of GSH-conjugated APAP metabolite levels, including APAP^GSH^, APAP^Cys^, and APAP^Nac^, were markedly reduced in *HuR*^Hep−/−^ mice 2 h after APAP overdose, whereas levels of the major non-GSH-dependent metabolites, APAP^Gluc^ and APAP^Sulf^, were comparable between genotypes. In parallel, RNA-seq analysis revealed reduced mRNA expression of multiple Gsts, including *Gsta1*, *Gsta2*, *Gsta4*, *Gstm6*, and *Gstm7*, in *HuR*^Hep−/−^ mouse livers. Although evidence supporting a dominant role for Gsts in APAP metabolism remains limited, the contribution of specific Gst isoforms to APAP-induced hepatotoxicity has been partially explored. For instance, *Gstp1/2*- and *Gstm*-deficient mice are resistant to APAP-induced hepatotoxicity despite exhibiting largely intact APAP metabolism (Arakawa et al. 2012; Henderson et al. 2000). In human cohorts, loss-of-function variants in *GSTT1* have been associated with improved outcomes following APAP overdose (Buchard et al. 2012). While these observations suggest that inhibition of certain Gst isoforms may be protective in specific contexts, substantial variability exists in Gst substrate specificity (Eaton and Bammler 1999). Moreover, although members of the Gstα family can catalyze GSH conjugation to NAPQI *in vitro* (Coles et al. 1988), the in vivo role of Gstα family in APAP-induced hepatotoxicity remains poorly defined. A limitation of the present study is that we did not determine the protein levels of specific Gst isoforms due to the lack of well-validated isoform-specific antibodies for mouse Gst proteins. Future studies will therefore focus on characterizing Gst isoform expression and enzymatic activity following APAP overdose, as well as quantifying APAP metabolites in the liver and bile of *HuR*^Hep−/−^ mice to more precisely define the contribution of HuR-dependent metabolic regulation to APAP detoxification.

In the present study, hepatocyte HuR emerged as a dynamic and multifaceted regulator of mitochondrial biology. Enhanced liver injury in *HuR*-deficient livers following APAP overdose was associated with pronounced alterations in mitochondrial morphology, dysregulated mitochondrial dynamics, impaired respiratory function, and increased mitochondrial permeabilization. Previous studies have highlighted diverse roles for HuR in regulating mitochondrial biology. For example, liver-specific *HuR* deficiency increases susceptibility to diet-induced MASLD by reducing expression of electron transport chain components, including cytochrome c (*Cycs*), NADH:ubiquinone oxidoreductase subunit B6 (*Ndufb6*), and ubiquinol–cytochrome c reductase binding protein (*Uqcrb*) (Zhang et al. 2020). In HEK293 cells, HuR maintains mitochondrial DNA integrity by facilitating cytoplasmic translocation of the long noncoding RNA, RNA processing endoribonuclease (*RMRP*) (Cascajo et al. 2016). while in differentiating B cells, mitochondrial energy production depends on HuR-mediated pre-mRNA processing of dihydrolipoamide S-succinyltransferase (*Dlst*) (Diaz-Munoz et al. 2015). HuR has also been shown to protect against mitochondrial oxidant stress through regulation of stress-responsive genes such as heme oxygenase 1 (Dery et al. 2020). Consistent with these reports, we have demonstrated that *HuR*-deficient livers exhibited reduced expression of both the mitochondrial fission protein Drp1 and the fusion protein Mfn2 following APAP overdose, accompanied by the appearance of large, elongated mitochondria at the 2-hour time point. Mechanistically, we identified Drp1 and Mfn2 as direct HuR targets. These findings are supported by prior in vitro work demonstrating that HuR binds the 3′ untranslated region of *Drp1* mRNA and that HuR loss promotes mitochondrial elongation (Bae et al. 2019). Interestingly, *HuR*-deficient hepatocytes from both untreated and APAP-exposed livers contained mitochondria with a more circular morphology. Increased mitochondrial circularity is commonly associated with loss of mitochondrial membrane potential and mitochondrial dysfunction following APAP exposure (Umbaugh et al. 2021). Reflecting cumulative defects in mitochondrial structure and function, release of mitochondrial intermembrane space proteins was markedly increased in *HuR*^Hep−/−^ livers at both 2 and 6 h after APAP overdose. Because release of mitochondrial endonucleases is a key driver of nuclear DNA fragmentation and hepatocyte necrosis (Bajt et al. 2006), these findings implicate mitochondrial dysfunction as a central contributor to the heightened liver injury observed in *HuR*^Hep−/−^ mice. HuR has been reported to regulate mitophagy through upregulation of PARKIN and BNIP3L (Yu et al. 2021), and inhibition of autophagy has been observed in male *HuR*-deficient livers following APAP overdose (Lu et al. 2024). Given that autophagic clearance of damaged mitochondria is a critical protective mechanism in APAP-induced liver injury (Chao et al. 2018; Ni et al. 2012), and that large, swollen mitochondria accumulated in *HuR*^Hep−/−^ hepatocytes at 24 h post-overdose, future studies should determine whether HuR directly regulates mitophagy during APAP-induced hepatotoxicity. A limitation of the TEM analysis in this study is the reliance on a single representative animal per group at each time point. As a result, mitochondrial features were quantified at the organelle level within individual animals, which may not fully capture inter-animal variability or allow robust statistical inference. Accordingly, these findings should be interpreted with caution, and future studies with increased biological replication and appropriate hierarchical statistical analysis will be necessary to confirm these observations quantitatively.

Sex differences in susceptibility to APAP-induced hepatotoxicity are well established, with female mice generally exhibiting greater resistance to APAP-induced liver injury compared with males. These differences have been attributed to several factors, including faster recovery of hepatic glutathione levels, reduced mitochondrial oxidant stress, and differences in antioxidant responses following APAP exposure (Du et al., 2014). A limitation of the present study is that all mechanistic experiments were performed in male mice. In our group’s preliminary experiments using female WT and *HuR*^Hep−/−^ mice treated with a higher APAP dose (350 mg/kg) to account for their greater resistance to APAP toxicity, we observed that hepatocyte HuR also appears to exert a protective role in female mice. However, because these experiments were conducted at a different APAP dose and sex-specific differences in APAP toxicity are well recognized, inclusion of these data would complicate direct comparisons with the male datasets presented here. Therefore, the current study focuses on male mice, and a separate study investigating sex-specific mechanisms of HuR in APAP-induced hepatotoxicity in females is currently in preparation. Future studies directly comparing male and female responses will be important to determine whether HuR regulates APAP detoxification and mitochondrial stress responses in a sex-dependent manner.

Compensatory liver regeneration, characterized by peri-necrotic hepatocyte proliferation, is essential for recovery following APAP overdose (Bhushan and Apte, 2019). In an incremental dose model using C57BL/6 J male mice, a moderate APAP dose (300 mg/kg) induces robust liver regeneration at 24 h, whereas a higher dose (600 mg/kg) results in severe liver injury accompanied by delayed regeneration and cell cycle arrest (Bhushan et al. 2014). In the present study, neither WT nor *HuR*^Hep−/−^ male mouse livers exhibited PCNA-positive staining at 24 h following treatment with 200 mg/kg APAP. Given the extensive hepatic necrosis and markedly elevated serum ALT levels observed in both genotypes at this time point, it is likely that 200 mg/kg APAP represents a non-regenerative dose in C57BL/6 N male mice. Although the use of a non-regenerative APAP dose limited direct assessment of liver regeneration, our findings nonetheless identify multiple mechanisms, including dysregulated APAP metabolism and mitochondrial dysfunction, through which hepatocyte-specific *HuR* deficiency exacerbates APAP-induced hepatotoxicity in male mice. We noticed that in the present study hepatocyte HuR does not completely prevent APAP-induced hepatotoxicity but rather appears to attenuate the severity of early injury. This observation is consistent with the role of HuR as a stress-responsive RNA-binding protein that regulates adaptive cellular responses by stabilizing transcripts involved in antioxidant defense, metabolism, and stress signaling. Because APAP-induced hepatotoxicity results from multiple converging mechanisms, including NAPQI formation, mitochondrial oxidative stress, and necrotic cell death, it is unlikely that induction of a single regulatory factor such as HuR would provide complete protection under conditions of severe overdose. Instead, our data support the interpretation that HuR contributes to protective stress-response pathways that help limit the extent of hepatocellular injury and functions as part of a broader adaptive stress-response network during APAP-induced liver injury.

## Supplementary Material

Supplementary material

## Data Availability

Data will be made available on request.

## Appendix A. Supporting information

Supplementary data associated with this article can be found in the online version at doi:10.1016/j.tox.2026.154480.
