# Supplementary material for: Dysregulation of xenobiotic metabolism and mitochondrial dysfunction exacerbate acetaminophen-induced hepatotoxicity in human antigen R-deficient male mice
Source: Toxicology. Author manuscript; Available in PMC 2026 May 27. (PMC13202677; doi:10.1016/j.tox.2026.154480)
Supplement: Supplementary material [file NIHMS2172149-supplement-Supplementary_material.docx]

# Supplementary table S1:

| *Mouse gene name* | Sequence |
| --- | --- |
| *Ccl2* | F: 5’- TTAAAAACCTGGATCGGAACCAA-3’  R: 5’- GCATTAGCTTCAGATTTACGGGT-3’ |
| *Gsta4* | F: 5’- TGATTGCCGTGGCTCCATTTA-3’  R: 5’- CAACGAGAAAAGCCTCTCCGT-3’ |
| *Gstm6* | F: 5’- TTGGAGAACAGGGTCATGGAC-3’  R: 5’- GGGTTCAAACATTCGATGCTGA-3’ |
| *Elavl1* | F: 5’- GGATGACATTGGGAGAACGAAT-3’  R: 5’- TGTCCTGCTACTTTATCCCGAA-3’ |
| *Gapdh* | F: 5’- AGGTCGGTGTGAACGGATTTG-3’  R: 5’- TGTAGACCATGTAGTTGAGGTCA-3’ |
| *Gclc* | F: 5’- GGGGTGACGAGGTGGAGTA-3’  R: 5’- GTTGGGGTTTGTCCTCTCCC-3’ |
| *Gclm* | F: 5’- AGGAGCTTCGGGACTGTATCC-3’  R: 5’- GGGACATGGTGCATTCCAAAA-3’ |
| *Hprt1* | F: 5’- CGTCGTGATTAGCGATGATGA-3’  R: 5’- CACACAGAGGGCCACAATGT-3’ |
| *Drp1* | F: 5’- CAAGGTTTTCTCGCCCAACG -3’  R: 5’- CTGCCCTTACCATCTGGATCTA -3’ |
| *Mfn2* | F: 5’- CTGGGGACCGGATCTTCTTC -3’  R: 5’- CTGCCTCTCGAAATTCTGAAACT -3’ |
| *Nfe2l2* | F: 5’- TCTTGGAGTAAGTCGAGAAGTGT-3’  R: 5’- GTTGAAACTGAGCAAAAAAGGC-3’ |
| *Tnfa* | F: 5’- CGTGGAACTGGCAGAAGAG-3’  R: 5’- ACAAGCAGGAATGAGAAGAGG-3’ |

**Supplementary Figure 1.** Caspase 3 cleavage was not detected in mouse livers following APAP-overdose. Proteins isolated from male livers collected at indicated time points following 200 mg/kg APAP were analyzed by Western blot. n=3 mice per group. Abbreviations: Caspase 3-CP, Caspase3 cleavage product.

**Supplementary Figure 2.** H&E-stained images of selected regions of interest (ROIs) used for FTIR analysis. Three ROIs located near the central vein were selected per animal, with three animals per group for spectral evaluation.

**Supplementary Figure 3.** Hepatocyte-specific *HuR* knockout does not alter electron transport chain (ETC) complex I-V protein expression but affects mitochondrial morphology and size. **(A)** Western blot analysis of mitochondrial OXPHOS complex proteins isolated from livers of WT and *HuR*^Hep-/-^. Protein levels were normalized to GAPDH and presented as fold change relative to the control groups. Data are presented as mean ± SEM, n = 4-6 mice per group. *p < 0.05, **p < 0.01. **(B)** Quantification of mitochondrial circularity and surface area of individual mitochondria in livers from WT and *HuR*^Hep-/-^ under untreated conditions and at 2, 6, and 24 hours following 200 mg/kg APAP administration. Data are presented as mean ± SEM; each symbol represents an individual mitochondrion. *p < 0.05, **p < 0.01.

**Supplementary Figure 4.** Overexpression HuR in Hepa 1-6 cells and validation of HuR targets by Ribonucleoprotein immunoprecipitation (RNP-IP). (A) Hepa1-6 cells were transfected with pcDNA RFP-HuR expression plasmid for 24 hours, with pcDNA RFP used as the vector control. Cells were treated in triplicate and whole cell lysates were analyzed by Western blot. n = 3 per group. (B) RNP-IP was performed in Hepa1-6 cells using anti-HuR or isotype IgG control antibodies, followed by qPCR analysis. Enrichment of target transcripts in HuR immunoprecipitates relative to IgG controls was calculated after normalization to the corresponding input samples. Data are presented as mean ± SEM. Statistical differences between two groups were analyzed using an unpaired two-tailed Student’s t-test. RNA-IP experiments were independently repeated twice using cells from separate culture dishes to confirm reproducible HuR binding.
